# High degree of polyclonality hinders somatic mutation calling in lung brush samples of COPD cases and controls

**DOI:** 10.1038/s41598-019-56618-1

**Published:** 2019-12-27

**Authors:** Gian-Andri Thun, Sophia Derdak, Francesc Castro-Giner, Katherine Apunte-Ramos, Lidia Águeda, Matthias Wjst, Anne Boland, Jean-François Deleuze, Umme Kolsum, Marion S. Heiss-Neumann, Adam Nowinski, Dorota Gorecka, Jens M. Hohlfeld, Tobias Welte, Christopher E. Brightling, David G. Parr, Antje Prasse, Joachim Müller-Quernheim, Timm Greulich, Mariarita Stendardo, Piera Boschetto, Imre Barta, Balázs Döme, Marta Gut, Dave Singh, Loems Ziegler-Heitbrock, Ivo G. Gut

**Affiliations:** 1 CNAG-CRG, Centre for Genomic Regulation (CRG), Barcelona Institute of Science and Technology (BIST), Barcelona, Spain; 2 Helmholtz-Zentrum München, National Research Centre for Environmental Health, Institute of Lung Biology and Disease, Neuherberg, Germany; 3 Institute of Medical Statistics, Epidemiology and Medical Informatics, Technical University Munich, Munich, Germany; 4 Centre National de Recherche en Génomique Humaine (CNRGH), Institut de Biologie François Jacob, CEA, Université Paris-Saclay, Evry, France; 5 University of Manchester, Manchester University NHS Foundation Trust, Manchester, UK; 6 EvA Study Center, Helmholtz-Zentrum München, Gauting, Germany; 7 2nd Department of Respiratory Medicine, National Institute of Tuberculosis and Lung Diseases, Warsaw, Poland; 8 Fraunhofer Institute for Toxicology and Experimental Medicine, Member of the German Center of Lung Research, Hannover, Germany; 9 Department of Respiratory Medicine, Hannover Medical School, Member of the German Center of Lung Research, Hannover, Germany; 10 Department of Infection, Immunity and Inflammation, Institute for Lung Health, University of Leicester, Leicester, UK; 11 Department of Respiratory Medicine, University Hospitals Coventry and Warwickshire NHS Trust, Coventry, UK; 12 Department of Pneumology, University Medical Center, Freiburg, Germany; 13 Department of Medicine, Pulmonary and Critical Care Medicine, University Medical Center Giessen and Marburg, Philipps-University, Marburg, Germany; 14 Department of Medical Sciences, University of Ferrara and University-Hospital of Ferrara, Ferrara, Italy; 15 Department of Pathophysiology, National Koranyi Institute for Pulmonology, Budapest, Hungary; 16 Department of Tumorbiology, National Koranyi Institute for Pulmonology, Budapest, Hungary; 17 Universitat Pompeu Fabra, Barcelona, Spain

**Keywords:** Genome, Chronic obstructive pulmonary disease

## Abstract

Chronic obstructive pulmonary disease (COPD) is induced by cigarette smoking and characterized by inflammation of airway tissue. Since smokers with COPD have a higher risk of developing lung cancer than those without, we hypothesized that they carry more mutations in affected tissue. We called somatic mutations in airway brush samples from medium-coverage whole genome sequencing data from healthy never and ex-smokers (n = 8), as well as from ex-smokers with variable degrees of COPD (n = 4). Owing to the limited concordance of resulting calls between the applied tools we built a consensus, a strategy that was validated with high accuracy for cancer data. However, consensus calls showed little promise of representing true positives due to low mappability of corresponding sequence reads and high overlap with positions harbouring known genetic polymorphisms. A targeted re-sequencing approach suggested that only few mutations would survive stringent verification testing and that our data did not allow the inference of any difference in the mutational load of bronchial brush samples between former smoking COPD cases and controls. High polyclonality in airway brush samples renders medium-depth sequencing insufficient to provide the resolution to detect somatic mutations. Deep sequencing data of airway biopsies are needed to tackle the question.

## Introduction

Exposure to cigarette smoke is the major risk factor for both lung cancer and chronic obstructive pulmonary disease (COPD)^1^. COPD diagnosis predates as many as 70% of lung cancer cases^2^, but the frequent co-existence is not only due to common causative agents. In fact, COPD also seems an independent risk factor for lung cancer as positive associations remain after accounting for smoking and co-occurring respiratory diseases^3,4^. While chronic inflammation in the lung could explain the direct link between COPD and lung cancer^5^, mitochondrial dysfunction has also been proposed to be one of the driving mechanisms^6^. The suggestive higher burden of mitochondrial reactive oxygen species in cells of the respiratory system in COPD patients, shown e.g. for airway smooth muscle cells^7^, could result in increased mutagenesis^8^. The same consequence may also be attributed to the downregulation of DNA repair genes in COPD cases^9^. An elevated mutational load in cells of the respiratory tract may then increase the risk of impairing cell cycle control genes and eventually transform cells to the point of forming malignant clones with the propensity to develop bronchial carcinomas.

The approach for detecting the somatic mutational burden in a particular tissue is well established for cancer. The standard way is to carry out whole genome sequencing (WGS) of a tumour sample as well as of a sample from a reference tissue (usually blood) to at least intermediate read depth (≈10–40x, used synonymously with medium-coverage WGS) and then simultaneously search for differences between the data of the two tissues. Examples of published genome-wide mutational loads in non-cancerous tissue are however scarce^10–12^. This is mainly because the described approach has appeared less promising beyond cancer, since the presumed lack of cell populations of recent clonal origin and the usual presence of a variety of cell types in available bulk tissue samples may limit a particular mutation to very few cells and to stay hence below the detection threshold. Nevertheless, WGS of intermediate read depth proved suitable for somatic mutation calling (SMC) in prostate tissue distant to clinically overt tumours, whereby many mutations were found that were neither present in the tumour nor in the blood representing the germline genome^13^. This indicates clonal expansion adjacent to a tumour in morphological normal prostate tissue and has also been observed in several other tissues including lung^14^. Furthermore, the number of different clonal lineages in a tissue may also depend on age as was shown for blood in which an expansion of pre-cancerous clonal cell populations was detected in an essential part of the elderly population^15^.

Little is known about the distribution and division rate of progenitor cells in the lung epithelium of healthy individuals and COPD patients, and consequently about the existence of cell populations of recent clonal origin in this tissue. Data from smokers, however, have suggested intensified consolidation of clones in the airways of smokers^16^, potentially carrying a high burden of persistent mutagenic alterations. Similarly, hyperplasia of airway epithelial cells has been reported to be a typical feature in the early COPD pathogenesis^17^. Therefore, we leveraged medium-coverage WGS data of lower lobe epithelial lung tissue, isolated from airway brushings, and blood as reference tissue from 12 elderly individuals to identify somatic single-nucleotide mutations (SSMs) and compared the total mutational load with respect to smoking status (ex-smokers vs. never smokers) and presence of COPD (within ex-smokers). For a subset of ex-smokers, upper lobe brushings were also analysed in order to reveal temporal information of mutational development. Verification analysis of the mutation calls resulted in a surprisingly small set of confirmed SSMs arguing for a highly polyclonal cell mixture in brush samples.

## Methods

### Study sample and bronchoscopy

Subjects 1–9, including three COPD cases as well as three ex-smoking and three never smoking controls (Table 1), were recruited from the EvA (Emphysema vs. Airway disease) study^18^, all providing blood and bronchial brushings from a lower lobe of the cancer-free lung by bronchoscopy. COPD was diagnosed by post-bronchodilation spirometry with a ratio between forced expiratory volume in one second and forced vital capacity (FEV1/FVC) <0.7. The cellular material of bronchial brushings represented an area of ≈9 cm^2^ of the airways and contained over 90% airway epithelial cells. Subjects 10–12, one COPD case and two healthy ex-smokers, were later selected from the same cohort, providing each two lung brush samples, one from a lower and one from an upper lobe of the lung, in addition to blood (Table 1). A more detailed description of the selection of the individuals and the collection of lung brush samples is given in Supplementary Methods. All individuals provided informed consent, and ethical approval was obtained from the respective local Ethics Committees (Ethics Committee of Philipps-University of Marburg, Albert-Ludwigs-University of Freiburg, Medizinische Hochschule Hannover, all Germany; Medical Research Council Budapest, Hungary; Ethics Committee of the Province of Ferrara, Italy; Ethics Committee of the National Tuberculosis and Lung Diseases Research Institute Warszawa, Poland; and NHS Research Ethics Committee of Leicestershire, Northamptonshire and Rutland Research Ethics Committee 2, UK). The central approval was obtained from the Ethic Committee of the University Hospital Munich, document # 400-07. All methods were performed in accordance with the principles of the Declaration of Helsinki.

**Table 1:** Characteristics of the study population.

Subject	Status	Sex	Age, [y]	Pack years	FEV1/FVC, [%]	GOLD stage
1	Case	m	70	35	55.7	2
2	Matched FS	m	68	32	76.6	0
3	Case	m	66	36	42.7	3
4	Matched FS	m	56	37	78.7	0
5	Case	f	61	27	67.0	1
6	Matched FS	f	61	59	85.5	0
7	NS	m	57	0	87.2	0
8	NS	f	68	0	84.1	0
9	NS	f	62	0	85.0	0
10	Case	m	68	35	41.0	3
11	FS	m	73	54	76.4	0
12	FS	m	64	55	98.0	0

Subjects 1–9 were recruited at a first stage of the study with only one lung brush sample collected, subjects 10–12 were later added, providing two lung brush samples each. Pack years is the number of cigarette packs smoked by day multiplied by the number of years the person has smoked. A COPD case is defined by FEV1/FVC < 70%. The GOLD stage is a measure of magnitude of the airflow obstruction in cases (1: mild; 2: moderate; 3: severe). None of the subjects has smoked in the past two years. FS = former smoker, NS = never smoker, FEV1 = forced expiratory volume in one second, FVC = forced vital capacity.

### Sample preparation and whole genome sequencing

Details about the DNA extraction, library preparation, WGS and post-processing of sequencing data from blood and brush samples are given in Supplementary Methods. Sequence reads were mapped to Human Reference Genome (hg19/GRCh37 decoy) using the Genome Multitool Mapper 2 (GEM2)^19^. The average mean coverage across samples was 23.7 (Supplementary Table S1), showing better results for samples of blood compared to airway tissue of the lower lobe (26.7 vs. 19.9, P = 0.02) and for samples acquired at the second stage of the project as opposed to the first stage (28.8 vs. 21.2, P = 0.005, Wilcoxon Rank-Sum tests). Since most SMC tools require a minimal positional read depth of ≈10 in both the affected and the reference tissue in order to evaluate the presence of a SSM, we defined the SMC power as the number of bp with a read depth of ≥10 in both tissues. Except for subject 2, whose brush sample was of substandard quality, the SMC power exceeded 2 Gb for all participants (Supplementary Table S1). We checked pair-wise relatedness (VCFtools v.0.1.12) between blood and lower lobe airway tissue based on germline variants called with SAMtools v.0.1.19^20^. All values were between 0.344 (subject 2) and 0.450 (subjects 11 and 12), leaving only subject 2 marginally below the recommended threshold for identity (0.354). The lower lobe brush sample of subject 10 showed unusual coverage peaks in *IGHM* and the presence of reads from Epstein-Barr virus (EBV) as is further described in Supplementary Methods and Supplementary Fig. S1.

### Strategy of somatic mutation calling

SMC was done on processed files of aligned sequence data (BAM format) after first ruling out the presence of large copy number alterations (ploidy deviations from 2) with Control-FREEC^21^. We applied three of the most popular SMC tools for cancer, all in standard mode: MuTect, v.1.1.4^22^, Strelka, v.1.0.14^23^ and VarScan 2, v.2.3.2^24^ and only concentrated on SSM calls. Rather than independently calling variants in affected and reference tissue against a reference genome, all three tools process the sequencing data of the two given tissues simultaneously and search for positions where the reference tissue matches the reference genome (variant allele frequency, VAF, ≈0) and the affected tissue shows an elevated VAF. Unlike tools jointly modelling genotypes and therefore requiring VAFs close to 0.5 or 1.0 to detect SSMs, MuTect and Strelka jointly model allele frequencies and are therefore considered particularly appropriate to call low-frequency SSMs^25^. In MuTect’s core algorithm a somatic variant model must fit better than models assuming artefacts or heterozygous SNPs as the reason for the presence of variant alleles. Strelka calls SSMs based on probabilities that VAFs differ in the two tissues and that the reference tissue has a homozygous genotype as in the reference genome. VarScan 2 uses a heuristic approach applying thresholds for the number and frequency of the variant allele in order to be called a germline variant and separates SSMs with the help of statistical tests comparing the affected with the reference tissue. In this case, the accuracy of calling low-frequency SSMs depends on the careful selection of a minimal VAF threshold in the affected tissue, which in turn is influenced by the available sequencing depth. Importantly, the availability of merely medium-coverage sequence data implied that mutations needed to be present in a substantial fraction of the cells in the sample of interest in order to be detectable (e.g. VarScan 2 required a VAF ≥ 0.15 in the applied setting). Further details about the performance of the SMC tools in benchmarking studies and the applied settings are given in Supplementary Methods. Finally, the tools also differ in the application and stringency of built-in filters that evaluate scores of base and read quality, of mapping quality of reads as well as for strand bias of alleles, which may all be related to sequencing or mapping errors. Post-calling filters can also be used to reduce the influence of such errors, but in order to avoid defining thresholds for post-calling filters, we chose to intersect the individual SSM results provided by each caller. Rather than unifying the results or considering calls detected by at least two of the three callers, we chose this conservative strategy in order to end up with a small, but presumably precise set of true positives. Intersections are represented with euler*APE*^26^ in area-proportional Venn-diagrams using ellipses. In individuals recruited at the second stage, similarity between the calls from upper and lower lobe brushings was assessed with the Dice similarity coefficient, S_Dice_ = 2*C/(N1 + N2), where C is the intersection between N1 and N2.

### Call annotation

Resulting SSM calls were annotated with dbSNP v.137^27^ and snpEff v3.6^28^. The UCSC Table Browser^29^ was used to determine overlap with DNase I hypersensitive sites (DHS, track: DNase Clusters) and repetitive regions (track: RepeatMasker). Structural annotation was based on ENSEMBL considering one category per call. Intermutation distances (IMDs) were used to determine clustering of SSM calls and were represented in Rainfall-plots with the help of in-house R scripts.

### Validation of the calling strategy

We validated our calling strategy by applying the same mapping and SMC tools and versions to a medulloblastoma (MB) and chronic lymphocytic leukemia (CLL) case, comparing for both a cancer sample with ≈40x WGS to a blood sample with ≈30x WGS data. These two cases had already been extensively investigated in a benchmarking study of different SMC pipelines^30^ and curated sets of verified mutations in the malignant samples were available. Calculation of sensitivity (recall) and precision (positive predictive value) of different SMC procedures was solely based on the predicted position (without considering mutation type). We further assessed the impact of a filtering script to the calls of VarScan 2 as recommended by the authors^31^. As the combination of the mapping software and the SMC tool had sometimes proven crucial for the overall accuracy of a pipeline^30^, we also carried out sensitivity analyses with GEM3, a meanwhile updated version of GEM2, as well as with the popular aligner BWA-mem^32^. We finally included MuTect 2, a completely revised version, which had in the meantime been integrated in GATK v.4.x and had performed well in a benchmark study^33^. Recall-Precision scatter plots were built with the function “scatterplot” in the package “car” in R 3.3.1.

### Confidence of mutation calls

Estimation of the likelihood of a SSM call to represent a true mutation was based on the overlap with known common or low-frequent (minor allele frequency, MAF, ≥1%) single nucleotide polymorphisms (SNPs) and the localisation in a genomic region difficult to map. For the former criterion, we annotated against an updated version of dbSNP (v.147) with the help of UCSC Table Browser (track “Common SNPs(147)”, representing about 0.5% of the genome), while for the latter, tracks “RepeatMasker”, a filter that leaves only ≈45% of the genome marked as non-repetitive, and “Mappability” (table “CRG Align 50”, a filter for uniqueness in a 50-bp window that assigns 77% of the genome to unique) were used. A call was set to high confidence if it did not coincide with a SNP, did not fall into a repetitive region and was unique based on the 50mer mappability filter. Medium-confidence assignment required the same SNP criterion, but respective positions could either be in a non-unique or repetitive region. The coincidence of a SSM call with a SNP or with a position in a non-unique as well as repetitive region built the low-confidence segment. We did not include in those confidence definitions positions coinciding with rare SNPs or SNPs with unknown frequencies in population-based studies due to potential unverified entries in recent versions of dbSNP (the track “All SNPs(147)” assigns over 5% of the genome to a polymorphism). Neither did we include read depth nor VAF filters for either of the tissues, as all three mutation callers already consider these parameters.

### Experimental verification of mutation calls

From each confidence segment, sets of 30 positions were selected randomly, but with the fix inclusion of all calls pointing either to non-synonymous SSMs or effecting a splice site (based on ENSEMBL, N = 4) and the requirement of at least one call from each individual being present. Calls lying on the Y-chromosome (N = 9) were not considered. Details about the primer design, the polymerase chain reactions (PCR) for amplifying the 90 selected positions, the concatenation with subsequent sequencing and data processing are given in Supplementary Methods. Four PCRs failed and successful concatenation of products of the remaining 86 reactions was confirmed by gel electrophoresis for each sample (length above 2 kb). After sequencing the concatenated products, SAMtools v.1.2 (mpileup) was used to determine coverage (upper limit: 150,000) as well as allele frequencies at positions of the targeted SSM calls (orphan reads were allowed and base quality scores of ≥30 were required). For each sample, VAF values at each call position were converted to a presence-absence matrix according to the following pattern. VAFs below 1% supported the reference allele. VAFs between 20 and 80% suggested heterozygous positions and those over 80% homozygous positions for the variant allele. VAFs between 5 and 20% at positions with good coverage (>1000) suggested the subclonal presence of the variant allele (and therefore possibly a SSM). All other VAFs (i.e. those between 1 and 5%, as well as those between 5% and 20% at positions with suboptimal coverage) were manually inspected, and decision whether the value supported the reference or the variant allele was taken according to results for the same position in other samples. The number of confirmed mutations was extrapolated to account for the total number of SSM calls per confidence category. Confidence of the non-verified SSM calls in the highest category was then re-assessed by utilizing three additional dichotomized features, which were read depth in reference and affected tissue (≥20 in both tissues vs. rest), VAF in reference tissue (>0 vs. 0), as well as presence of a SNP, either rare or of unknown frequency, at the respective position (UCSC Table Browser track “All SNPs(147)”, but not “Common SNPs(147)”).

### Statistical analysis

Statistical calculations were carried out with STATA 11.

## Results

### Somatic mutation calling

**Figure 1:**
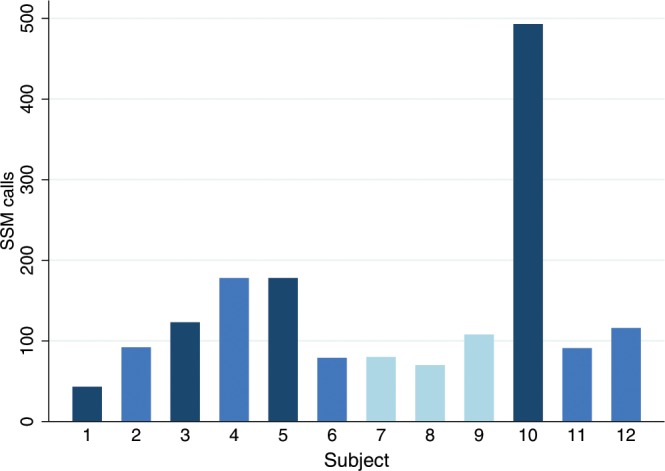
Numbers of somatic single-nucleotide mutation (SSM) calls in lower lobe bronchial brushings per subject. Numbers represent the intersected results of three somatic mutation calling tools. Shades of blue represent COPD cases (dark), ex-smokers without COPD (azure) and never smokers (light).

We observed large differences between the individual results of the three SMC tools. While VarScan 2 and MuTect called more than 2800 SSMs in the lower lobe lung brush sample of each individual, the unfiltered outcome of Strelka was one order of magnitude smaller. Although similar in size, the overlaps between the two permissive calling tools did not coincide with the results of the restrictive caller, resulting in very small intersections between the three callers (Supplementary Fig. S2). Overlapping SSM calls ranged from 43 (subject 1) to 493 (subject 10) and amounted to a total of 1651 (1645 unique ones) when only considering lower lobe samples (Fig. 1). Strikingly, neither brushings from COPD patients, nor those from ex-smokers with normal lung function did unambiguously show elevated numbers of SSM calls when compared to those of never smokers. Subject 10, already mentioned for some unusual sequencing coverage at immunoglobulin loci (Supplementary Methods and Supplementary Fig. S1A), was the only individual with a clearly increased number of SSMs in the intersected results. Moreover, IMDs were particularly low in this sample, an issue far less notable in the brush sample of the upper lobe of the same individual (Supplementary Fig. S3) or in any other brush sample. Although clustering of mutations (kataegis) is a well-described phenomenon in certain cancers^34^, the observed pattern favoured a technical problem since the clustering was distributed over the whole genome. Accordingly, similarities between intra-individual calls in upper and lower lobe airway brushings were smaller in this subject than in the other two with this type of data (Supplementary Table S2). Nevertheless, we decided not to exclude the deviant sample at this stage owing to two reasons. First, the increased number and unusual distribution of calls could have been related to a local EBV infection (Supplementary Methods and Supplementary Fig. S1B), which can enhance mutational loads^35^. Second, comparing the uncorrected number of SSM calls would only be informative if the SMC power was equal between the subjects. When comparing the numbers relative to the positions covered by enough reads to determine the presence of SSMs, subject 10 was no longer an outlier. More importantly, this adjustment did not alter the main counter-intuitive finding, namely that there were no clear differences between the two groups of former smokers (with and without COPD) and never smokers in terms of the total somatic mutational burden suggested by state-of-the-art SMC tools (Table 2).

**Table 2:** Number of somatic single-nucleotide mutation calls per Mb in lower lobe lung brushings of COPD cases and controls. Genome size was calculated based on positions with read depth ≥10 in both, reference and affected tissue. Ranges are given due to the small number of individuals per category.

	N	MuTect	VarScan 2	Strelka	Intersection
COPD cases	4	1.97–3.00	2.26–9.03	0.10–0.54	0.02–0.19
former smokers	5	1.36–12.17	1.79–5.00	0.14–0.95	0.03–0.12
never smokers	3	2.01–2.81	1.91–7.94	0.18–0.25	0.03–0.04

### Validation of the applied strategy in cancer

In order to validate the applied strategy to call SSMs, we carried out the same procedure with two cancer cases (one CLL and one MB case) from which we had fully verified somatic mutation call sets^30^. The strategy of taking the intersection of the individual results of the three callers proved accurate (Supplementary Fig. S4A). Compared to intersections of only two tools, the loss in recall was marginal and clearly compensated by a material gain in precision. Replacing the aligner GEM2 with the newer version GEM3 improved results for Strelka and respective intersections in the MB data set, but no such effect was found in the CLL data set. Further sensitivity analyses with BWA-mem confirmed that the intersection strategy of three tools led to higher precision than alternative strategies, while the recall remained high (Supplementary Fig. S4B). Improvements from MuTect to MuTect 2 were clearly visible in the individual results, but were not essential in intersections. Therefore, we did not consider applying these updated mapping or calling tools to the sequencing data of the brushings (EvA data set).

### Critical appraisal of the somatic mutation calls

**Figure 2:**
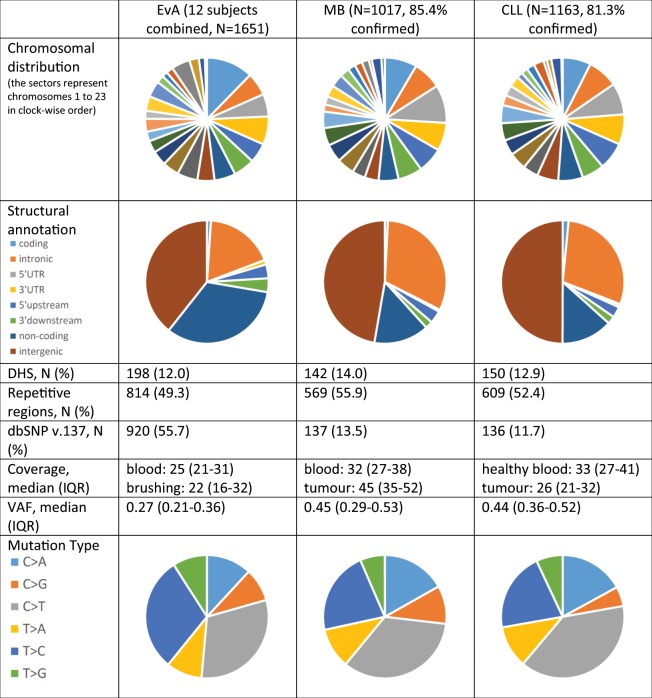
Characteristics of somatic single-nucleotide mutation calls in lung brushings of subjects from the EvA study (N = 12) as well as in tumour tissue of two cancer cases based on the same calling procedure. Coverages and VAFs were based on the Strelka results. MB = medulloblastoma, CLL = chronic lymphocytic leukemia, DHS = DNase I hypersensitive sites, IQR = interquartile range, VAF = variant allele frequency.

Despite the successful validation of our SMC strategy in cancer cases, we further assessed some characteristics of the 1651 suggested SSMs in lower lobe brushings across all subjects and compared them with those of the (true and false positive) calls in the two cancer cases when applying the same tools. Calls did not show material differences in terms of chromosomal distribution, structural annotation, overlap with DHS or repetitive regions, as well as mutation type (Fig. 2). As 9 of the 12 individuals in the EvA data set were former smokers, a higher proportion of C > A substitutions in lung epithelial tissue (as compared to malignant brain or blood tissue) could have been anticipated^34^, but sample size was obviously too limited to draw conclusions from the absence of such an association. There were other features of the calls in the EvA data set that deviated considerably. Variant alleles peaked at markedly lower frequencies (median VAF ranged from 0.22 for subject 12 to 0.35 for subject 2) than in MB (0.45) or CLL (0.44). This is not unexpected since clonal heterogeneity is likely much higher in non-cancerous samples. However, results from the two cancer cases suggested that the reliability of calls with decreased VAF was reduced. The false positive proportion of the SSM calls shared between the three callers showed median VAFs of 0.23 (MB) and 0.29 (CLL), whereas the true positive calls barely deviated from 0.5 (median VAFs of 0.47 and 0.46 for MB and CLL, respectively). Furthermore, median coverage of the calls in the EvA data set was lower, and for only 53.1% of the called SSMs, coverage at the respective position was at least 20 in both, the reference and the affected tissue. Respective numbers in the cancer cases were 92.9% (MB) and 77.9% (CLL). The feature creating most doubts about the certainty of calls in the EvA data set was, however, the high overlap with polymorphisms in dbSNP v.137 (55.7%). This was markedly higher than for calls from the MB (13.5%) and CLL sample (11.7%), and the latter proportions even decreased further when including only the confirmed calls (to 5.3% and 4.6%, respectively). Especially at suboptimal sequencing depth of the respective position in blood, the chance for a heterozygous SNP to appear absent in blood, but not in lung (which would hence result in a wrong SSM call), may be substantial. We defined then three confidence levels for our calls based on location in genomic regions difficult to assess (i.e. high repetitivity or low mappability) or of polymorphisms in an updated version of dbSNP (Methods). 21.9% of the 1651 SSM calls overlapped with a common or low-frequent SNP. This is lower than stated in Fig. 2 as only SNPs with a MAF > 1% were considered here. Only 14.8% of calls were neither in a repetitive nor in a non-unique region, while 33.3% were in both. Taken together, less than 10% of the suggested SSMs (N = 156) were assessed as high confidence, whereas N = 711 and N = 784 were assigned to the middle- and low-confidence category. Finally, in the three individuals from which upper and lower lobe brushings were available, ≈15–20% of the SSM calls were shared between the two sampled locations (Supplementary Table S2). While such calls may appear more robust (mutational events that occurred early in lung development), they can also be explained by the aforementioned uncertainty of correctly assigning the reference allele to the blood sample, which could lead to faulty calls in both, upper and lower lobe brushings, at once. Indeed, only four of the 46 calls in this group were classified as highly confident (≈9%).

### Verification of selected mutation calls

The 90 selected calls (30 for each confidence category) are shown in Supplementary Table S3. For four positions representing calls in the high- (N = 1), medium- (N = 1) and low-confidence (N = 2) category, no functioning PCR could be established. The sequencing depth of the remaining positions in the verification approach is shown in Supplementary Table S4 across all 27 tissue samples. One position did not show any coverage in any of the samples and was excluded as a faulty primer pair not designed to include the position of the respective SSM call was used. In general, we achieved very high coverage for the selected positions in the concatenated amplicons (overall median coverage was ≈27,000), but due to the design of the experiment, median coverage per position over the tissue samples varied strongly (minimum of 87, maximum of >150,000). Although positional coverages of up to 100 (red colour) or 1000 (orange) may be considered of limited value for a verification attempt, it is noteworthy that we detected reads for the remaining 85 candidate positions across all 27 samples, meaning that products of all PCRs had been present in the final ligations. This includes those from samples with low concentration of DNA stock solution (subject 4, bronchial tissue) or for which DNA from former library preparations had to be amplified due to missing stock solution (lower lobe bronchial tissue of subjects 1, 2 and 12, Supplementary Methods). There were only very few positions at which we could not assess the likelihood of the presence of a variant allele, and these did not concern the subjects in which the mutation was actually suspected.

Overall, VAF results for many positions did not support the presence of a variant allele in the brushings of the respective individuals, indicating considerable false positive calls in the WGS-based SMC results (Table 3 and more detailed in Supplementary Tables S5 to S7). Grouped by confidence category, this finding accounted for 21 of 28 (75.0%), 21 of 29 (72.4%) and 12 of 28 (42.9%) of SSM calls included in the verification analysis. There was only one case where verification of the SSM call seemed successful, showing a clearly higher VAF in the brush than in the blood sample at the respective position (Supplementary Table S5, position 5 in subject 6). For other positions, the detected VAF in the respective sample was slightly elevated, but either limited coverage or comparisons with the same position in samples not suspected to carry the mutation nevertheless favoured the exclusive presence of the reference allele (N = 1, 6 and 3 for high-, medium- and low-confidence calls, respectively; indicated by a red 0 in Supplementary Tables S5 to S7). Germline heterozygous variants are expected to exhibit VAFs very close to 0.5 in blood and airway brushings as, unlike for SSMs, neither polyclonality nor infiltration of other cell types reduces this frequency. Not surprisingly, we detected such cases (indicated by a black 1) particularly in the low-confidence category (N = 9, 32.1%) as calls overlapping with common and low-frequent SNPs had been classified there. Furthermore, we observed several cases where results for the respective position in the targeted sample supported the subclonal presence of a variant allele, but those findings were not tissue-specific (N = 5, 1 and 4 for high-, medium- and low-confidence calls, respectively; indicated as a red or green 1). They may either point to allele-specific sequencing or mapping bias, especially if deviant VAFs were present in samples of several individuals, or to postzygotic mosaicism if only detected in one individual. The 84 non-verified calls (out of 85 with valid verification results) also included three calls that were made in lower and upper lobe brushings of the same individual. We note here that even very high coverage observed in the verification analysis did sometimes not prevent us from results which were difficult to interpret. As even calls in the high-confidence category proved faulty, we retrospectively assessed more stringent criteria for defining a high-confidence call. Of the 27 high-confidence calls that could not be verified, 9 would have been down-graded if a WGS read depth filter of 20 in both, blood and brush sample, had been applied, 5 would not have met the requirement of 0 variant alleles in blood, and 10 would not have been considered if overlap with any entry in dbSNP v.147 (i.e. even if of unknown MAF in the general population) had been taken into account. Only 7 out of 28, including the one that was verified, would have ended up in the high confidence category if such still rather permissive criteria had been applied in a combined way.

**Table 3:** Verification analysis of selected somatic single-nucleotide mutation (SSM) calls, stratified by confidence category. Only one of 85 SSM calls, targeted for verification, could be confirmed. WGS = whole genome sequencing.

Verification result of variant allele	high-confidence (N = 28)	medium-confidence (N = 29)	low-confidence (N = 28)	Interpretation of original WGS call
found in target tissue (subject-specific)	1	0	0	correct
found in target and reference tissue (subject-specific or non-specific)	5	1	4	mosaicism (if subject-specific verification); allele-specific sequencing bias
fully present in target and reference tissue (≈50 or 100%)	0	1	9	single nucleotide polymorphism
unlikely or unclearly present (e.g. few high-quality reads)	1	6	3	position difficult to call
missing in target and reference tissue	21	21	12	sequencing or mapping inaccuracies

In summary, the verification analysis could only confirm one predicted SSM. Extrapolating this result would leave us with only a handful true SSMs of the 1651 calls, leaving over 99% as presumably false positives. High confidence in the overlapping output of common SMC tools is only warranted if stringent requirements in terms of sequencing coverage, complexity of the region and overlap with data bases of polymorphisms are met.

## Discussion

Motivated by the fact that several studies called numerous somatic mutations in healthy tissue by sequencing affected and reference tissue to an intermediate read depth^13,14^, we applied here a similar approach for the genome-wide calling of SSMs in airway brushings from COPD cases and controls. The number of included individuals was obviously small and at best appropriate to formulate a trend respective to a possible difference in mutation numbers between cases and controls, but the strong assumption of detecting at least differing numbers between former and never smoking controls served as a proof-of-concept of the methodology. However, we were unable to detect a robust number of SSMs in this study, neither in lung brushings of former smokers in general, nor specifically in COPD patients. While we first seek evidence for a biological interpretation of this observation, we concentrate later on limitations of the applied methodology and discuss alternative ways to tackle this task.

As it has never been investigated in a genome-wide manner, it could be argued that, years after smoking cessation, negative selection of strongly mutated lung progenitor cells eventually leads to a harmonization of mutational levels between former and never smokers, which would explain our failure to detect such a difference in samples of airway epithelial cells. There is, however, compelling evidence for a higher number of accumulated mutations in the airways of ex-smokers. First, investigations of microsatellite instability in former smokers at selected chromosomal sites support such a notion^36^. Second, the mutational load in tumours of (current and former) smokers is clearly elevated compared to tumours of never smokers^37,38^. Third, the risk of developing lung cancer in former smokers never reverts to the level of never smokers in epidemiological studies^39,40^, which could be due to a greater number of persistent mutations and hence a higher likelihood to affect cancer driver genes. Moreover, the complete failure to detect any mutational mark in airway epithelial cells of brush samples does not match available results from other tissues. Estimates of 600 SSMs for blood^10^ and a few thousands for sun-exposed epidermis^41^ were reported. Numbers for internal organs often derive from tumour-adjacent tissue and also lie in the range of several hundred^13,14^. Data from lung tumours suggest around 10 per Mb in smokers^32^, a similar load in former smokers^38^ and one order of magnitude fewer in never smokers^37^. Since a large part of the mutational burden is assumed to be present before tumour initiation^42^, we may expect cumulative SSM numbers in the range of hundreds in samples of airway epithelial cells of elderly never smokers, similar to blood or internal organs. Such a number would also be obtained by assuming an intrinsic mutation rate of about three per cell division^42^ and a cell division rate of around once per several months for airway epithelial cells^43^. With SSM estimates of 150 per year due to smoking one cigarette pack per day^37^, the mutational burden in bronchial tissue of former smokers may hence be well in the thousands. Taken together, methodological reasons must explain the lack of verifiable SSMs in our study.

In the absence of a standard approach how to estimate the genome-wide mutational load in healthy tissues, we selected here a procedure that was strongly based on established methods for tumours (medium-coverage WGS of bulk samples from target and reference tissue of the same individual). Three different callers were chosen due to the fact that concordance between different tools is sometimes low^25,30,33^. Our conservative strategy of only considering the overlap was believed to lead to a set with high precision as was confirmed in a validation approach with two cancer cases. The resulting number of SSM calls of around 100 per brush sample was indeed smaller than anticipated, but could have represented lower bounds due to the conservative strategy and the limited SMC power in several individuals. However, an assessment of the confidence of calls and a subsequent verification approach corroborated the suspicion of having picked up mainly false positives. Hence, stringent application of criteria like minimal read depth, permitted number of variant alleles in reads of the reference tissue and low-complex or blacklisted regions remain essential and cannot entirely be replaced by intersecting results from different callers in order to reduce false positives to a negligible number.

**Figure 3:**
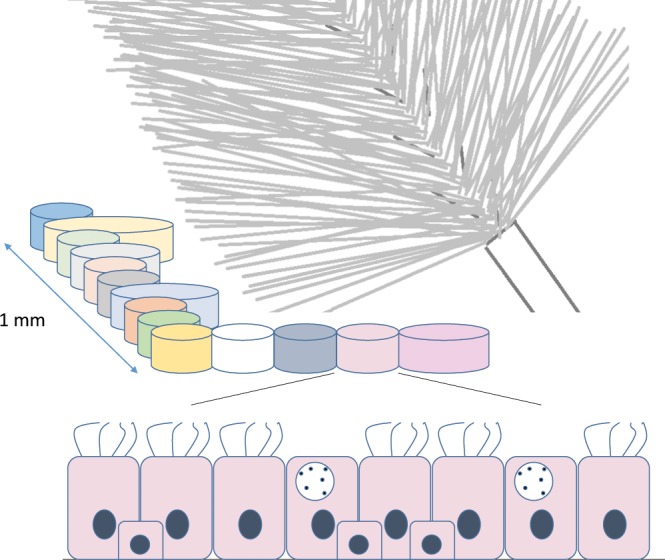
Representation of mutant clones in the bronchial epithelium. Coloured cylinders represent different cellular clones of recent origin in the airway epithelium, each containing cells that share the same somatic mutations. Such a clone (shown for the one in salmon at the bottom) may contain a mixture of cell types, such as differentiated secretory cells (shown with granules in the cytoplasm), ciliated cells (depicted with cilia at the outer surface) and unstratified basal cells (square shaped), all sitting on the basement membrane (line at the very bottom). Such a cellular arrangement is named pseudostratified columnar epithelium and is typical for the airways. Clone size may vary, but presumably lies in the sub-millimeter range, which is much smaller than the diameter and the length of a brush used in bronchoscopy (shown on the right). Consequently, a brush sample contains many different clones and the proportion of cells carrying the same somatic mutations is very small and falls below the detection limit. Successful somatic mutation calling requires sampling a smaller area (i.e. coming closer to the detection limit by harvesting a higher proportion of cells carrying the same somatic mutations) and/or sequencing to a higher read depth (i.e. lowering the detection limit).

A far more crucial issue is the large number of false negatives in this study. Intersecting results derived from different calling tools is admittedly prone to result in many false negatives in the sense that results can only become as sensitive as the least sensitive caller performs^33^. However, all three callers have shown reasonable sensitivity in benchmarking studies^44^ as well as in our validation with cancer data. The ability to detect a variant allele at a specific position in bulk samples depends on the proportion of reads supporting the variant. This proportion is related to the number of cells carrying the variant allele with respect to the total number of cells. Its minimal required value to reliably call the presence of a variant allele also varies according to the available read depth at that position (Fig. 3). In tumour biopsies, the relative number of cells sharing a particular mutation is generally high as recent clonal expansion has taken place, even in the case of a substantial amount of non-malignant tissue or intratumoural heterogeneity^45^. This becomes even more obvious when a minority clone outgrows other divergent clones, e.g. by positive selection owing to treatment. Consequently, read depth requirements are modest, and common SMC tools are accurate when using the typical minimal VAF of about 0.15 in order to call a mutation (i.e. ≈30% of the sampled cells must carry the mutation). For non-pathological tissue, it is generally unknown how many cells originate from a recent specific progenitor cell and form an ancestral cell lineage. In blood, depletion of cell lineages is common in the elderly^15^, and a sample of a centenarian suggested the presence of only two stem cell populations, which explains the ability to call hundreds of SSMs, albeit with high sequence coverage (60×)^10^. On the other hand, studies of selected genes in biopsies of the epidermis found clonal unit sizes per mutation of ≈0.2 mm^2^ (around 2000 cells)^41^ or even less^46^. Proportions of affected cells were so small that ultra-deep sequencing (500×) was necessary, a procedure which is still prohibitively costly for whole genomes. Interestingly, skin fibroblast samples contained SSMs with >0.1 VAF, but those may represent events that arose early in skin development^47^. Large clonal cell populations of recent origin must also be widespread in tumour-adjacent tissue of several internal organs, in order to explain the detection of several hundred SSMs that were neither in blood nor in the tumour close-by using medium-coverage sequencing^13,14^. The fact that biopsies were gained in close proximity to tumour tissue may, however, point to field effects, i.e. the presence of molecular abnormalities in tissue that appears histologically normal. The airway epithelium contains facultative progenitor cells that mainly proliferate in response to injury^48^, which could be smoking- or COPD-related. Indeed, smokers have shown higher basal cell division rates leading to clonal consolidation^16^, and basal cell hyperplasia is one of the earliest lesions in the pathogenesis of COPD^17^. In addition, and unlike lung biopsies^10,49^, brush samples contain very small quantities of immune cells^50^ (confirmed in our cytospins), all enhancing the chance of finding mutations confined to reasonably large cell populations.

The observed absence of basically any SSMs in our brush samples point however to major limitations of our approach. While biopsies typically originate from areas (sections) of 1–5 mm^2^ ^13,41,47^, brushings have a >100x higher and less cohesive area sampled (Fig. 3 and Supplementary Methods). Assuming similar sizes of clonal units, even ultra-deep sequencing approaches^41^ may struggle to compensate for this loss of resolution. In terms of COPD, we must further acknowledge that by random sampling of one location in the airways, we may not necessarily have hit a spot severely affected by the disorder. Furthermore, it is conceivable that clonal sizes due to pre-cancerous metaplastic processes increase into a more detectable range in later stages of the disease or with very old age. If recently expanded clones are absent and mutations thus only confined to few cells, even small biopsies could be insufficient to detect a robust number of them. Such an interpretation underlies most likely the finding of no^10^ or very few SSMs^51^ in post-mortem brain samples with sequencing depth up to 100×. The very few detected mutations could represent early events similar to our study, in which their occurrence was most likely limited to the small time window in embryonic lung development after the separation of mesodermal (forming components of the blood) and endodermal tissue (forming epithelia, e.g. that of the respiratory tract). The few overlapping mutations between brushings from upper and lower lobe lung within the same individual would also belong to this type (assuming successful verification). In contrast, for SSMs appearing postnatally, including all smoking-related ones, brush sampling, in spite of its easy execution, is an inadequate detection strategy, at least in combination with medium-coverage sequencing where about 30% of sampled cells are required to be affected.

Recently, more sophisticated methods to find rare SSMs in healthy tissues emerged^52,53^. Single adult stem cells, or single differentiated cells via the detour of reprogramming them into induced pluripotent stem cells (iPSC)^47^, can be amplified in culture, and consequently mutations become clonally expanded as well. WGS at intermediate read depth is then sufficient to detect them because their VAFs lie very close to 0.5 unless representing events during cell proliferation in culture. Even post-mitotic neurons could be assessed by amplification of single-cell DNA before application of WGS^12^. Several studies using such novel methods have reported unexpected findings. Small differences in mutation rates between tissues of quiescent organs and high-turnover tissues were for instance reported by independent methods^11,52^. This challenges the concept backed by tumour data that cell division rate is a better indicator of the mutational load in a tissue than age^34,54,55^. Mutational loads seems also higher than suggested from sequencing bulk tissue samples although stochastic effect can play a role if single cells are analysed individually. For example, genetically reprogrammed skin fibroblasts from children contained on average over 1000 SSMs in spite of sampling locations not exposed to the sun, and only few could be attributed to the reprogramming process^47^. Postnatally non-dividing neurons also showed several hundred mutations already one year after birth and further SSM accumulation correlated with age during adulthood^12^. Amplification artifacts and the high error rate have so far been considered major limitations of single-cell WGS, but as results were in good agreement with those using other novel methodology^52,56^, the accumulation of SSMs must also occur spontaneously or transcription-associated. Taking these observations together, it is fair to assume that the mutational load in airway epithelial single cells of elderly people lies well above 1000, even in the absence of former or current smoking exposure, and the need to standardize procedures is obvious.

In summary, it remains elusive why cigarette smokers that retain normal lung function are at lower risk of developing lung cancer than those who develop airflow obstruction. In this work, serving as a pilot study by tackling this question from a mutational point of view, the mutational load in samples of bronchial epithelial cells could not be accurately estimated due to sizes of clonal cell populations below the detection limit of the applied methodology. The synthesis with results from similar work further implies that i) sequencing to an intermediate read depth is rarely sufficient to illuminate the mutational load in samples of healthy tissue; ii) airway brushing by bronchoscopy is an inappropriate sampling strategy given the relatively large area from which cells are gained, possibly not even surmountable by deep-sequencing data; iii) tissue directly adjacent to cancer does not serve as a valuable proxy for healthy tissue as the cell lineage composition might resemble more closely that of a tumour. Consequently, the question whether COPD influences lung cancer risk via the accumulation and persistence of somatic mutations^8^ remains up for debate. Although the typically non-reversible processes in the lung of COPD patients like hypoxia, inflammation, tissue damage and remodeling indeed represent conditions favorable to mutagenesis, they may also act on the better survival of emerging tumour cells. For instance, COPD-related symptoms seem to leave methylation signatures on immune genes^57^ and lung cancer associated genes^58^, which may in turn facilitate tumour growth in a manner independent of mutagenesis. The mutational burden in bronchial epithelial tissue is maybe currently best estimated via amplifying single stem cells in organoid cultures. Such approaches may help to decipher whether differences of the somatic mutational burden in airway epithelial tissue between ex-smokers and never smokers as well as between COPD patients and non-obstructive individuals with similar smoking history explain disparity in lung cancer risk, a question that remains intriguing.

## Supplementary information

Supplementary Information.

## Data Availability

Processed sequencing data from this study have been deposited in BAM-format at the European Genome-phenome archive (EGA) under accession number EGAS00001003406. Additional information for reproducing the results is available upon reasonable request.
