# Supplementary material for: High degree of polyclonality hinders somatic mutation calling in lung brush samples of COPD cases and controls
Source: Sci Rep. 2019 Dec 27;9:20158. doi: 10.1038/s41598-019-56618-1 (PMC6934450; doi:10.1038/s41598-019-56618-1)
Supplement: Supplementary file 1 — Supplementary Information. [file 41598_2019_56618_MOESM1_ESM.docx]

**High degree of polyclonality hinders somatic mutation calling in lung brush samples of COPD cases and controls**

Gian-Andri Thun^1^, Sophia Derdak^1^, Francesc Castro-Giner^1^, Katherine Apunte-Ramos^1^, Lidia Águeda^1^, Matthias Wjst^2,3^, Anne Boland^4^, Jean-François Deleuze^4^, Umme Kolsum^5^, Marion S. Heiss-Neumann^6^, Adam Nowinski^7^, Dorota Gorecka^7^, Jens M. Hohlfeld^8,9^, Tobias Welte^9^, Christopher E. Brightling^10^, David G. Parr^11^, Antje Prasse^9,12^, Joachim Müller-Quernheim^12^, Timm Greulich^13^, Mariarita Stendardo^14^, Piera Boschetto^14^, Imre Barta^15^, Balázs Döme^16^, Marta Gut^1,17^, Dave Singh^5^, Loems Ziegler-Heitbrock^6^, Ivo G. Gut^1,17*^

* Corresponding author:

Ivo G. Gut, ivo.gut@cnag.crg.eu

Centro Nacional de Análisis Genómico, Baldiri Reixac 4, Barcelona Science Park - Tower I,

08028 Barcelona, Spain

**Authors’ affiliations:**

1 CNAG-CRG, Centre for Genomic Regulation (CRG), Barcelona Institute of Science and Technology (BIST), Barcelona, Spain

2 Helmholtz-Zentrum München, National Research Centre for Environmental Health, Institute of Lung Biology and Disease, Neuherberg, Germany

3 Institute of Medical Statistics, Epidemiology and Medical Informatics, Technical University Munich, Munich, Germany

4 Centre National de Recherche en Génomique Humaine (CNRGH), Institut de Biologie François Jacob, CEA, Université Paris-Saclay, Evry, France

5 University of Manchester, Manchester University NHS Foundation Trust, Manchester, UK

6 EvA Study Center, Helmholtz-Zentrum München, Gauting, Germany

7 2nd Department of Respiratory Medicine, National Institute of Tuberculosis and Lung Diseases, Warsaw, Poland

8 Fraunhofer Institute for Toxicology and Experimental Medicine, Member of the German Center of Lung Research, Hannover, Germany

9 Department of Respiratory Medicine, Hannover Medical School, Member of the German Center of Lung Research, Hannover, Germany

10 Department of Infection, Immunity and Inflammation, Institute for Lung Health, University of Leicester, Leicester, UK

11 Department of Respiratory Medicine, University Hospitals Coventry and Warwickshire NHS Trust, Coventry, UK

12 Department of Pneumology, University Medical Center, Freiburg, Germany

13 Department of Medicine, Pulmonary and Critical Care Medicine, University Medical Center Giessen and Marburg, Philipps-University, Marburg, Germany

14 Department of Medical Sciences, University of Ferrara and University-Hospital of Ferrara, Ferrara, Italy

15 Department of Pathophysiology, National Koranyi Institute for Pulmonology, Budapest, Hungary

16 Department of Tumorbiology, National Koranyi Institute for Pulmonology, Budapest, Hungary

17 Universitat Pompeu Fabra, Barcelona, Spain

Supplementary Methods

*Selection of individuals*

We recruited a total of 12 individuals from the EvA (Emphysema vs. Airway disease) study, a multi-center project whose goal is to better understand different mechanisms leading to COPD. Participation in EvA was restricted to elderly ex-smokers with and without airflow limitation and a few never smoking controls. At a first stage of our study, nine individuals were selected, representing three COPD cases with different magnitude of airflow limitation, three former smokers with normal lung function, matched by sex, as well as age or smoking history, and three never smoking controls (Table 1, subjects 1-9). All those individuals provided non-cancerous airway epithelial tissue by bronchoscopy from one of the lower lobes of the lung as well as blood as reference tissue. At a second stage, three ex-smoking individuals (one case and two controls, subjects 10-12) were added, providing two lung brush samples each, one from a lower and one from an upper lobe of the lung, in addition to blood. A COPD case was defined by post-bronchodilation spirometry with a ratio between forced expiratory volume in one second and forced vital capacity (FEV1/FVC) below 0.7. Mild (GOLD stage 1) and moderate (GOLD stage 2) cases were defined by %-predicted FEV1 ≥80 and <80/≥50, respectively. Severe cases (GOLD stage 3) have corresponding values of <50/≥30 %-predicted FEV1. The time span since the last cigarette smoked was at least two years for all ex-smoking subjects.

*Collection and purity of lung brush samples*

Flexible bronchoscopy was carried out after premedication with a sedative and after local anesthesia in semi recumbent position. Protected brushes (Olympus BC-202D 5010) with a brush length of 10 mm and a diameter of 5 mm at bristle level were used to sample sub-segment airways of either the right lower or the right upper lobe. The brush was moved in and out three times and then rotated in the airway five times. Samples from three brushes used for three sub-segment airways were pooled. Given the dimensions of the brush and considering that it was moved back and forth within the airway by double its length, every brush covered ≈3 cm^2^, such that the material studied was derived from a bronchial surface of about 9 cm^2^. No bleeding was noted with the procedure. Cytospins of the material revealed airway epithelial cells plus less than 10% leukocytes.

*DNA extraction and library preparation*

DNA was extracted using Phenol-Chloroform for blood and AllPrep DNA/RNA Mini kit (Qiacube) for brush samples. The short-insert paired-end libraries were prepared with a PCR-free protocol. KAPA Library Preparation Kit (Kapa Biosystems) with NEXTflex PCR-Free Barcodes (Bioo Scientific) was used. In short, a pool of 0.2 to 1.7 μg of 90 region-specific PCRs of 27 Illumina libraries were used as an input. The amplified DNA was end-repaired, adenylated and ligated to Illumina-specific single indexed paired-end adaptors. The adaptor-modified end library was size-selected and purified with AMPure XP beads (Agencourt, Beckman Coulter) in order to eliminate any not ligated adaptors. The final libraries were quantified by Kapa Library Quantification Kit for Illumina platforms (Kapa Biosystems).

*Whole genome sequencing*

Libraries were sequenced using Illumina HiSeq2500 in paired-end mode with a read length of 2x101 bp using TruSeq SBS Kit v4. We generated a mean of 12 million paired reads of raw data in a fraction of a sequencing v4 flow cell lane, following the manufacturer’s protocol. Image analysis, base calling and quality scoring of the run were processed using the manufacturer’s software Real Time Analysis (RTA 1.18.66.3) and followed by generation of FASTQ sequence files by CASAVA. Sequence reads (>100 million pairs per sample) were mapped to Human Reference Genome (hg19/GRCh37 decoy) using the Genome Multitool Mapper 2 (GEM2). Uniquely aligning reads, ranging from 81.1% (subject 3, blood) to 85.5% (subject 11, lower lobe brushing), were cleaned from duplicates with Picard v.1.110, and the Genome Analysis Toolkit (GATK) v.3.1-1 was used for local realignment around indels. Mean coverage was calculated based on the unambiguous (non-N) portion of hg19 (2,858,674,665 bases).

*Sequencing peculiarities in one lung brush sample*

The lower lobe brush sample of subject 10 showed unusual coverage peaks in the constant part of the immunoglobulin heavy locus *IGHM* on 14q32.33 (Supplementary Fig. S1A) and to a lesser degree in the immunoglobulin kappa locus *IGK* on 2p11.2, an issue which appeared neither in the blood or upper lobe brush sample of the same individual, nor in any other individual. As only exonic regions were concerned, this could point to a contamination with RNA derived from B-cells. This sample was also the only one with positive results from the alignment against the genome of Epstein-Barr virus (EBV, genome NC_007605), which was included in our reference assembly. No viral reads were detected in the sample of the upper lobe lung tissue of the same patient. The number of total reads aligned to EBV was however low (≈1000) and the viral genome was not uniformly covered (Supplementary Fig. S1B), both arguing against a current (lytic) infection at this particular site. The reads may point to a former infection of airway epithelial cells (in agreement with the reported constant loss of extrachromosomal viral EBV DNA in proliferating cells [^1^](#_ENREF_21)) or may originate from infiltrating B-cells, the more common cell type for EBV infections. The latter would be in line with the finding that the affected individual suffered from advanced COPD (GOLD stage 3), and COPD progression is associated with the presence of B-lymphocytes in the small airways [^2^](#_ENREF_22).

*The somatic mutation calling tools*

We applied three of the most popular SMC tools for cancer: MuTect, v.1.1.4, Strelka, v.1.0.14 and VarScan 2, v.2.3.2. Strelka and MuTect scored well in benchmarking studies with cancer data when used individually ^3,^[^4^](#_ENREF_26), but also in combination [^5^](#_ENREF_27). The third tool, VarScan 2, showed very good sensitivity in detecting tumour mutations especially at high sequencing depth [^4,6^](#_ENREF_26). We concentrated in this study on SSMs as the applied MuTect version does not call somatic indel mutations (SIMs), and agreement of SIM calls between different tools is known to be much lower [^5^](#_ENREF_27). Owing to the lack of similar studies that have used these tools for SMC in non-cancerous tissues, we ran all tools in standard mode. For MuTect, we supplied dbSNP v.137 as well as the Catalogue of Somatic Mutations in Cancer (COSMIC) v.54 [^7^](#_ENREF_30), and output calls in variant call format (VCF). VarScan 2 was applied on data generated by mpileup from SAMtools, and protocols for SMC were followed as recommended by the producer [^8^](#_ENREF_31), but with a slightly more permissive minimal variant allele frequency (VAF) for the affected tissue (0.15 instead of 0.2). In the ideal case of a monoclonal sample, this value would be close to 0.5 for a heterozygous SSM (or 1.0 for a homozygous one), but such numbers are not expected due to different clonal cell populations and infiltration of a variety of cell types in the samples of the affected tissue. Additionally, sequencing and alignment errors can lead to background noise, which also influences the allelic distribution at a given base position.

*Primer design, amplification and sequencing for the verification of mutation calls*

We designed primers with Primer-BLAST within a window extending to 250 bp on both sides of the call. Default settings were used, but in some cases of medium- and low-confident calls, filters for low complexity or repetitive regions had to be relaxed. Primers had to be unique at the genome-level, but non-specific pairs were also tolerated if the predicted PCR products were of different length. If no such primer pair could be designed, the SSM call was replaced. Predicted amplicon sizes for the 90 positions were between 125 and 455 bp with primer lengths between 19 and 28 nucleotides and melting temperatures between 57.1 and 62.9 °C. Test DNA from K562 cells (leukemia cell line) was used to carry out the 90 individual PCRs (56 ng DNA, 0.2 mM dNTP, 0.1 μM of each primer, 3 mM MgCl_2_, 1x PCR buffer and 7 U *Taq* polymerase in 70 μL reaction volume) in order to assess the successful design of the primers (PCR: initialization at 95 °C / 10 min, 30 cycles with denaturation at 94 °C / 30 s, annealing 60 °C / 90 s and elongation 72 °C / 20 s). 86 reactions (95.6%) showed bands on agarose gels in the expected range. Product quality on gel electrophoresis (e.g. the presence of smears) was then used to adjust MgCl_2_ concentration (to 2 mM) and thermal profile (annealing time 60 s at 61 °C) resulting in the establishment of three groups of different PCR conditions pooling 38, 25 and 23 reactions. Interference of primer pairs in such defined multiplex PCRs, although visible on the gels, proved a minor issue, and hence according procedure was applied to DNA from all 12 blood and 15 brush samples. For one sample with insufficient DNA concentration (subject 4, brushing), we reduced the amount of required genetic material to 25 ng, and the same amount of DNA from former library preparations was used in those three brush samples (all from lower lobe lung) where no DNA stock solution was left (subjects 1, 2 and 12). For each sample, amplicons of the three pools were then purified and end-repaired, and 20 μL was used with 5 μL T4 DNA ligase and 25 μL ligation buffer to ligate the PCR products in random order (15 min at 20 °C). Successful concatenation of the products was confirmed by gel electrophoresis for each sample (length above 2 kb). KAPA Library Preparation Kit (Roche) was used to obtain libraries with fragment sizes of ≈400 bp length and concentrations of above 10 nM. Paired-end sequencing (2x 100 nucleotides read length) to a total of 10 M reads per sample was carried out on an Illumina HiSeq, followed by aligning to human reference genome hg19 with GEM3. Between 20.6 and 40.6% of the sequenced reads per sample could not be mapped pointing to reads covering the junctions between PCR products. Split-read mapping was not considered as reads covering breakpoints would usually not include the position of the SSM call, and unmapped reads were thus discarded.

1 Nanbo, A., Sugden, A. & Sugden, B. The coupling of synthesis and partitioning of EBV's plasmid replicon is revealed in live cells. *The EMBO journal* **26**, 4252-4262, doi:10.1038/sj.emboj.7601853 (2007).

2 Hogg, J. C. *et al.* The nature of small-airway obstruction in chronic obstructive pulmonary disease. *The New England journal of medicine* **350**, 2645-2653, doi:10.1056/NEJMoa032158 (2004).

3 Xu, H., DiCarlo, J., Satya, R. V., Peng, Q. & Wang, Y. Comparison of somatic mutation calling methods in amplicon and whole exome sequence data. *BMC genomics* **15**, 244, doi:10.1186/1471-2164-15-244 (2014).

4 Wang, Q. *et al.* Detecting somatic point mutations in cancer genome sequencing data: a comparison of mutation callers. *Genome medicine* **5**, 91, doi:10.1186/gm495 (2013).

5 Alioto, T. S. *et al.* A comprehensive assessment of somatic mutation detection in cancer using whole-genome sequencing. *Nature communications* **6**, 10001, doi:10.1038/ncomms10001 (2015).

6 Cai, L., Yuan, W., Zhang, Z., He, L. & Chou, K. C. In-depth comparison of somatic point mutation callers based on different tumor next-generation sequencing depth data. *Scientific reports* **6**, 36540, doi:10.1038/srep36540 (2016).

7 Forbes, S. A. *et al.* COSMIC: mining complete cancer genomes in the Catalogue of Somatic Mutations in Cancer. *Nucleic acids research* **39**, D945-950, doi:10.1093/nar/gkq929 (2011).

8 Koboldt, D. C., Larson, D. E. & Wilson, R. K. Using VarScan 2 for Germline Variant Calling and Somatic Mutation Detection. *Current protocols in bioinformatics* **44**, 15 14 11-17, doi:10.1002/0471250953.bi1504s44 (2013).

Supplementary Tables

| Subject | Coverage blood  (mean) | Coverage airway (lower lobe, mean) | SMC power  (lower lobe, Gb) | Coverage airway (upper lobe, mean) | SMC power  (upper lobe, Gb) |
| --- | --- | --- | --- | --- | --- |
| 1 | 19.1 | 14.1 | 2.07 |  |  |
| 2 | 27.2 | 7.6 | 0.80 |  |  |
| 3 | 32.0 | 17.4 | 2.34 |  |  |
| 4 | 37.8 | 12.7 | 2.04 |  |  |
| 5 | 22.2 | 19.3 | 2.45 |  |  |
| 6 | 21.6 | 15.7 | 2.31 |  |  |
| 7 | 23.6 | 20.7 | 2.44 |  |  |
| 8 | 25.9 | 23.6 | 2.57 |  |  |
| 9 | 23.6 | 17.4 | 2.43 |  |  |
| 10 | 25.3 | 24.9 | 2.57 | 23.2 | 2.58 |
| 11 | 31.0 | 30.5 | 2.73 | 30.8 | 2.73 |
| 12 | 30.8 | 35.1 | 2.74 | 28.0 | 2.71 |

**Supplementary Table S1**. Tissue-stratified mean coverages and somatic mutation calling (SMC) power for each subject of the study population. The SMC power was defined by the genomic size of positions that have a minimal read depth ≥10 in blood as well as in airway brush samples. Brushings of the upper lobe airways were only available for subjects 10 to 12.

|  | MuTect | | VarScan 2 | | Strelka | | Intersection | |
| --- | --- | --- | --- | --- | --- | --- | --- | --- |
| Subject | SSM calls  (lower; upper) | DC | SSM calls  (lower; upper) | DC | SSM calls  (lower; upper) | DC | SSM calls  (lower; upper) | DC |
| 10 | 5224; 3627 | 0.136 | 9856; 7227 | 0.135 | 1392; 457 | 0.058 | 493; 87 | 0.045 |
| 11 | 3713; 3665 | 0.175 | 8298; 8025 | 0.162 | 592; 613 | 0.141 | 91; 87 | 0.124 |
| 12 | 3875; 3333 | 0.184 | 8370; 8577 | 0.163 | 712; 537 | 0.146 | 116; 84 | 0.220 |

**Supplementary Table S2**. Number of somatic single-nucleotide mutation (SSM) calls in lower and upper lobe bronchial brushings and their intra-individual similarity, stratified by the somatic mutation calling tools and their intersection. Similarities were expressed by the Dice-coefficient (DC).

| High confidence | | | | Medium confidence | | | | Low confidence | | | |
| --- | --- | --- | --- | --- | --- | --- | --- | --- | --- | --- | --- |
| Nr | Chr. | Position | Subject | Nr | Chr. | Position | Subject | Nr | Chr. | Position | Subject |
| 1 | 1 | 160865804 | 10 | 31 | 1 | 19934742 | 10 | 61 | 1 | 146650363 | 10 |
| 2 | 1 | 210900153 | 8 | 32 | 1 | 77165856 | 10 | 62 | 1 | 234411511 | 10 |
| 3 | 2 | 126363667 | 2 | 33 | 1 | 89448729 | 10 | 63 | 2 | 134650758 | 12 |
| 4 | 2 | 144544369 | 11 | 34 | 1 | 121313146 | 4 | 64 | 3 | 655821 | 6 |
| 5 | 2 | 183180257 | 6 | 35 | 1 | 143157311 | 9 | 65 | 3 | 133442608 | 2 |
| 6 | 3 | 48421639 | 11 | 36 | 2 | 92076569 | 5 | 66 | 4 | 9035262 | 10 |
| 7 | 3 | 76255754 | 12 | 37 | 2 | 122678782 | 5 | 67 | 6 | 168563807 | 3 |
| 8 | 4 | 8176578 | 10 | 38 | 2 | 190788531 | 10 | 68 | 7 | 151940238 | 4 |
| 9 | 4 | 88813496 | 10 | 39 | 3 | 156071937 | 11 | 69 | 8 | 52059335 | 9 |
| 10 | 4 | 113325466 | 5 | 40 | 3 | 161147038 | 10 | 70 | 8 | 54244369 | 10 |
| *11* | *4* | *179856556* | *3* | 41 | 4 | 71347116 | 7 | 71 | 10 | 77340410 | 9 |
| 12 | 5 | 1562399 | 12 | 42 | 4 | 158306110 | 3 | 72 | 11 | 129550714 | 2 |
| 13 | 5 | 61529291 | 7 | 43 | 5 | 1588835 | 6 | 73 | 14 | 28309043 | 1 |
| 14 | 5 | 112314380 | 10 | 44 | 5 | 16040190 | 1 | 74 | 14 | 37349832 | 7 |
| 15 | 7 | 4768383 | 9 | 45 | 7 | 66489994 | 4 | 75 | 14 | 85878628 | 4 |
| 16 | 8 | 133360677 | 2 | 46 | 8 | 21387018 | 12 | 76 | 15 | 24496361 | 4 |
| 17 | 9 | 68410754 | 12 | *47* | *9* | *30692508* | *4* | 77 | 15 | 34916901 | 5 |
| 18 | 10 | 127580315 | 4 | 48 | 10 | 21716924 | 10 | 78 | 16 | 32513507 | 3 |
| 19 | 11 | 103193399 | 9 | 49 | 10 | 38569694 | 5 | *79* | *17* | *15465022* | *6* |
| 20 | 12 | 63359310 | 10 | 50 | 11 | 90015909 | 10 | 80 | 18 | 14861823 | 5 |
| 21 | 12 | 93058894 | 5 | 51 | 12 | 10253754 | 2 | 81 | 18 | 63567858 | 7 |
| 22 | 12 | 102971655 | 4 | 52 | 12 | 10659570 | 10 | 82 | 19 | 48462795 | 3 |
| 23 | 13 | 25671274 | 10 | 53 | 12 | 64115282 | 9 | 83 | 20 | 29606022 | 12 |
| 24 | 13 | 36043611 | 2 | 54 | 14 | 92719144 | 8 | 84 | 21 | 17766298 | 7 |
| 25 | 14 | 28292897 | 1 | 55 | 15 | 68449069 | 8 | 85 | 21 | 42824661 | 10 |
| 26 | 15 | 48194240 | 9 | 56 | 17 | 25287188 | 7 | 86 | 22 | 17382019 | 8 |
| 27 | 18 | 50295166 | 5 | 57 | 17 | 58095893 | 12 | *87* | *22* | *17497459* | *1* |
| 28 | 19 | 53727460 | 10 | 58 | 18 | 26964615 | 2 | 88 | 22 | 48465870 | 4 |
| 29 | 21 | 11026555 | 6 | 59 | 21 | 24405628 | 3 | 89 | X | 8387066 | 9 |
| 30 | 22 | 41460280 | 10 | 60 | 22 | 19780291 | 10 | 90 | X | 111506015 | 8 |

**Supplementary Table S3**. Selected somatic single-nucleotide mutation calls for the verification analysis according to confidence. The four calls shown in italics were excluded as individual PCR reactions failed to generate any product.

11-100

101-1000

1001-10,000

10,001-100,000

>100,000

Up to 10

**Supplementary Table S4**. Coverage in the verification analysis across samples at positions of suspected mutations (N=86). The first 29 positions represent high-confidence calls, followed by 29 medium- and 28 low-confidence calls. Position 11 was excluded (design of wrong primer pairs). Columns are ordered by subject and blood (bl) and brush (br) sample. Brush samples belonging to the same subject are indicated by lower lobe (brL) and upper lobe (brU). Samples with low coverage (columns 2, 4, 8 and 26) could be attributed to low-concentration or absent DNA stock solution (Supplementary Methods).

| Subject | | 1 | | 2 | | 3 | | 4 | | 5 | | 6 | | 7 | | 8 | | 9 | | 10 | | 11 | | 12 | |
| --- | --- | --- | --- | --- | --- | --- | --- | --- | --- | --- | --- | --- | --- | --- | --- | --- | --- | --- | --- | --- | --- | --- | --- | --- | --- |
| Sample | | Bl | Br | Bl | Br | Bl | Br | Bl | Br | Bl | Br | Bl | Br | Bl | Br | Bl | Br | Bl | Br | Bl | Br | Bl | Br | Bl | Br |
| 1 | 160865804 | 0 | 0 | 0 | 0 | 0 | 0 | 0 | 0 | 0 | 0 | 0 | 0 | 0 | 0 | 0 | 0 | 0 | 0 | 0 | 0,0 | 0 | 0,0 | 0 | 0,0 |
| 1 | 210900153 | 0 | 0 | 0 | 0 | 0 | 0 | 0 | 0 | 0 | 0 | 0 | 0 | 0 | 0 | 1 | 1 | 0 | 0 | 0 | 0,0 | 1 | 1,1 | 0 | 0,0 |
| 2 | 126363667 | 1 | 1 | 1 | 1 | 0 | 0 | 1 | 1 | 1 | 1 | 0 | 0 | 1 | 1 | 1 | 1 | 0 | 0 | 0 | 0,0 | 0 | 0,0 | 0 | 0,0 |
| 2 | 144544369 | 0 | 0 | 0 | nd | 0 | 0 | 0 | 0 | 0 | 0 | 0 | 0 | 0 | 0 | 0 | 0 | 0 | 0 | 0 | 0,0 | 0 | 0,0 | 0 | 0,0 |
| 2 | 183180257 | 0 | 0 | 0 | 0 | 0 | 0 | 0 | 0 | 0 | 0 | 0 | 1 | 0 | 0 | 0 | 0 | 0 | 0 | 0 | 0,0 | 0 | 0,0 | 0 | 0,0 |
| 3 | 48421639 | 1 | 1 | 1 | 1 | 1 | 1 | 1 | 1 | 0 | 0 | 1 | 1 | 0 | 0 | 1 | 1 | 1 | 1 | 0 | 0,0 | 1 | 1,1 | 1 | 1,1 |
| 3 | 76255754 | 1 | 1 | 0 | 0 | 0 | 0 | 1 | 1 | 1 | 1 | 1 | 1 | 1 | 1 | 0 | 0 | 1 | 1 | 1 | 1,1 | 1 | 1,1 | 1 | 1,1 |
| 4 | 8176578 | 0 | 0 | 0 | nd | 0 | 0 | 0 | nd | 0 | 0 | 0 | 0 | 0 | 0 | 0 | 0 | 0 | 0 | 0 | 0,0 | 0 | 0,0 | 0 | 0,0 |
| 4 | 88813496 | 0 | 0 | 0 | 0 | 0 | 0 | 0 | 0 | 0 | 0 | 0 | 0 | 0 | 0 | 0 | 0 | 0 | 0 | 0 | 0,0 | 0 | 0,0 | 0 | 0,0 |
| 4 | 113325466 | 0 | 0 | 0 | 0 | 0 | 0 | 0 | 0 | 0 | 0 | 0 | 0 | 0 | 0 | 0 | 0 | 0 | 0 | 0 | 0,0 | 0 | 0,0 | 0 | 0,0 |
| 5 | 1562399 | na | na | na | na | na | na | na | na | na | na | na | na | na | na | na | na | na | na | na | na | na | na | na | na |
| 5 | 61529291 | 0 | 0 | 0 | 0 | 0 | 0 | 0 | 0 | 0 | 0 | 0 | 0 | 0 | 0 | 0 | 0 | 0 | 0 | 0 | 0,0 | 0 | 0,0 | 0 | 0,0 |
| 5 | 112314380 | 0 | 0 | 0 | 0 | 0 | 0 | 0 | 0 | 0 | 0 | 0 | 0 | 0 | 0 | 0 | 0 | 0 | 0 | 0 | 0,0 | 0 | 0,0 | 0 | 0,0 |
| 7 | 4768383 | 0 | 0 | 0 | 0 | 0 | 0 | 0 | 0 | 0 | 0 | 0 | 0 | 0 | 0 | 0 | 0 | 0 | 0 | 0 | 0,0 | 0 | 0,0 | 0 | 0,0 |
| 8 | 133360677 | 0 | 0 | 0 | 0 | 0 | 0 | 0 | 0 | 0 | 0 | 0 | 0 | 0 | 0 | 0 | 0 | 0 | 0 | 0 | 0,0 | 0 | 0,0 | 0 | 0,0 |
| 9 | 68410754 | 0 | 1 | 0 | 1 | 0 | 0 | 0 | 0 | 0 | 0 | 0 | 0 | 0 | 0 | 0 | 0 | 0 | 0 | 0 | 0,0 | 0 | 0,0 | 0 | 0,0 |
| 10 | 127580315 | 0 | 0 | 0 | 0 | 0 | 0 | 1 | 1 | 0 | 0 | 0 | 0 | 0 | 0 | 0 | 0 | 0 | 0 | 0 | 0,0 | 0 | 0,0 | 0 | 0,0 |
| 11 | 103193399 | 0 | 0 | 0 | 0 | 0 | 0 | 0 | 0 | 0 | 0 | 0 | 0 | 0 | 0 | 0 | 0 | 0 | 0 | 0 | 0,0 | 0 | 0,0 | 0 | 0,0 |
| 12 | 63359310 | 0 | 0 | 0 | 0 | 0 | 0 | 0 | 0 | 0 | 0 | 0 | 0 | 0 | 0 | 0 | 0 | 0 | 0 | 0 | 0,0 | 0 | 0,0 | 0 | 0,0 |
| 12 | 93058894 | 0 | 0 | 0 | 0 | 0 | 0 | 0 | 0 | 0 | 0 | 0 | 0 | 0 | 0 | 0 | 0 | 0 | 0 | 0 | 0,0 | 0 | 0,0 | 0 | 0,0 |
| 12 | 102971655 | 1 | 1 | 0 | 0 | 0 | 0 | 0 | 0 | 0 | 0 | 0 | 0 | 0 | 0 | 0 | 0 | 0 | 0 | 0 | 0,0 | 0 | 0,0 | 0 | 0,0 |
| 13 | 25671274 | 0 | 0 | 0 | 0 | 0 | 0 | 0 | 0 | 0 | 0 | 0 | 0 | 0 | 0 | 0 | 0 | 0 | 0 | 0 | 0,0 | 0 | 0,0 | 0 | 0,0 |
| 13 | 36043611 | 0 | 0 | 0 | 0 | 0 | 0 | 0 | 0 | 0 | 0 | 0 | 0 | 0 | 0 | 0 | 0 | 0 | 0 | 0 | 0,0 | 0 | 0,0 | 0 | 0,0 |
| 14 | 28292897 | 0 | 0 | 0 | 0 | 0 | 0 | 0 | 0 | 0 | 0 | 0 | 0 | 0 | 0 | 0 | 0 | 0 | 0 | 0 | 0,0 | 0 | 0,0 | 0 | 0,0 |
| 15 | 48194240 | 0 | 0 | 0 | 0 | 0 | 0 | 0 | 0 | 0 | 0 | 0 | 0 | 0 | 0 | 0 | 0 | 0 | 0 | 0 | 0,0 | 0 | 0,0 | 0 | 0,0 |
| 18 | 50295166 | 0 | 0 | 0 | 0 | 0 | 0 | 0 | 0 | 0 | 0 | 0 | 0 | 0 | 0 | 0 | 0 | 0 | 0 | 0 | 0,0 | 0 | 0,0 | 0 | 0,0 |
| 19 | 53727460 | 0 | 0 | 0 | 0 | 0 | 0 | 0 | 0 | 0 | 0 | 0 | 0 | 0 | 0 | 0 | 0 | 0 | 0 | 0 | 0,0 | 0 | 0,0 | 0 | 0,0 |
| 21 | 11026555 | 0 | 0 | 0 | 0 | 0 | 0 | 0 | 0 | 0 | 0 | 0 | 0 | 0 | 0 | 0 | 0 | 0 | 0 | 0 | 0,0 | 0 | 0,0 | 0 | 0,0 |
| 22 | 41460280 | 0 | 0 | 0 | 0 | 0 | 0 | 0 | 0 | 0 | 0 | 0 | 0 | 0 | 0 | 0 | 0 | 0 | 0 | 0 | 0,0 | 0 | 0,0 | 0 | 0,0 |

**Supplementary Table S5**. Verification results of high-confidence somatic single-nucleotide mutation calls. Yellow background colour shows the subject (column) and chromosomal position (row) at which the mutation was suspected. For each subject, verification results are shown for blood (bl) and lung brush (br) samples, the latter is further divided into one from lower lobe followed by one from upper lobe (subjects 10 to 12). The mutation was always suspected in lower lobe brushings unless at chromosome 9, position 68410754, where it was predicted at both airway locations. Numbers in black give clear indication of the absence (variant allele frequency, VAF ≈0) or presence of one (VAF ≈0.5) variant allele at the respective position. Red colour indicates less certainty and the decision about absence or presence was not only taken by the VAF, but also by read depth and cross-sample comparisons at the respective position (see Methods). Green colour suggests a high likelihood of subclonal presence of one variant allele, but may also point to positions difficult to call if present across several subjects. na=not available, nd=not determinable.

| Subject | | 1 | | 2 | | 3 | | 4 | | 5 | | 6 | | 7 | | 8 | | 9 | | 10 | | 11 | | 12 | |
| --- | --- | --- | --- | --- | --- | --- | --- | --- | --- | --- | --- | --- | --- | --- | --- | --- | --- | --- | --- | --- | --- | --- | --- | --- | --- |
| Sample | | Bl | Br | Bl | Br | Bl | Br | Bl | Br | Bl | Br | Bl | Br | Bl | Br | Bl | Br | Bl | Br | Bl | Br | Bl | Br | Bl | Br |
| 1 | 19934742 | 0 | 0 | 0 | 0 | 0 | 0 | 0 | 0 | 0 | 0 | 0 | 0 | 0 | 0 | 0 | 0 | 0 | 0 | 0 | 0,0 | 0 | 0,0 | 0 | 0,0 |
| 1 | 77165856 | 0 | 0 | 0 | 0 | 0 | 0 | 0 | 0 | 0 | 0 | 0 | 0 | 0 | 0 | 0 | 0 | 0 | 0 | 0 | 0,0 | 0 | 0,0 | 0 | 0,0 |
| 1 | 89448729 | 0 | 0 | 0 | 0 | 0 | 0 | 0 | 0 | 0 | 0 | 0 | 0 | 0 | 0 | 0 | 0 | 0 | 0 | 0 | 0,0 | 0 | 0,0 | 0 | 0,0 |
| 1 | 121313146 | 1 | 1 | 1 | 0 | 1 | 1 | 1 | 0 | 1 | 1 | 1 | 1 | 1 | 1 | 1 | 1 | 1 | 1 | 1 | 1,1 | 1 | 1,1 | 1 | 1,1 |
| 1 | 143157311 | 1 | 1 | 0 | 0 | 0 | 0 | 1 | 1 | 1 | 1 | 1 | 1 | 1 | 1 | 1 | 1 | 1 | 1 | 0 | 0,0 | 1 | 1,1 | 1 | 1,1 |
| 2 | 92076569 | 0 | 0 | 0 | 0 | 0 | 0 | 0 | 0 | 0 | 0 | 0 | 0 | 0 | 0 | 0 | 0 | 0 | 0 | 0 | 0,0 | 0 | 0,0 | 0 | 0,0 |
| 2 | 122678782 | 0 | 0 | 0 | 0 | 0 | 0 | 0 | 0 | 0 | 0 | 0 | 0 | 0 | 0 | 0 | 0 | 0 | 0 | 0 | 0,0 | 0 | 0,0 | 0 | 0,0 |
| 2 | 190788531 | 0 | 0 | 0 | 0 | 0 | 0 | 0 | 0 | 0 | 0 | 0 | 0 | 0 | 0 | 1 | 1 | 0 | 0 | 0 | 0,0 | 0 | 0,0 | 0 | 0,0 |
| 3 | 156071937 | 0 | 0 | 0 | 0 | 0 | 0 | 0 | 0 | 0 | 0 | 0 | 0 | 0 | 0 | 0 | 0 | 0 | 0 | 0 | 0,0 | 0 | 0,0 | 0 | 0,0 |
| 3 | 161147038 | 0 | 0 | 0 | 0 | 0 | 0 | 0 | 0 | 0 | 0 | 0 | 0 | 0 | 0 | 0 | 0 | 0 | 0 | 0 | 0,0 | 0 | 0,0 | 0 | 0,0 |
| 4 | 71347116 | 0 | 0 | 0 | 0 | 0 | 0 | 0 | 0 | 0 | 0 | 0 | 0 | 0 | 0 | 0 | 0 | 0 | 0 | 0 | 0,0 | 0 | 0,0 | 0 | 0,0 |
| 4 | 158306110 | 0 | 0 | 0 | 0 | 0 | 0 | 0 | 0 | 0 | 0 | 0 | 0 | 0 | 0 | 0 | 0 | 0 | 0 | 0 | 0,0 | 0 | 0,0 | 0 | 0,0 |
| 5 | 1588835 | 0 | 0 | 0 | 0 | 0 | 0 | 0 | 0 | 0 | 0 | 0 | 0 | 0 | 0 | 0 | 0 | 0 | 0 | 0 | 0,0 | 0 | 0,0 | 0 | 0,0 |
| 5 | 16040190 | 0 | 0 | 0 | 0 | 0 | 0 | 0 | 0 | 0 | 0 | 0 | 0 | 0 | 0 | 0 | 0 | 0 | 0 | 0 | 0,0 | 0 | 0,0 | 0 | 0,0 |
| 7 | 66489994 | 0 | 0 | 0 | 0 | 0 | 0 | 0 | 0 | 0 | 0 | 0 | 0 | 0 | 0 | 0 | 0 | 0 | 0 | 0 | 0,0 | 0 | 0,0 | 0 | 0,0 |
| 8 | 21387018 | 0 | 0 | 0 | 0 | 0 | 0 | 0 | 0 | 0 | 0 | 0 | 0 | 0 | 0 | 0 | 0 | 0 | 0 | 0 | 0,0 | 0 | 0,0 | 0 | 0,0 |
| 10 | 21716924 | 0 | 0 | 0 | 0 | 0 | 0 | 0 | 0 | 0 | 0 | 0 | 0 | 0 | 0 | 0 | 0 | 0 | 0 | 1 | 1,1 | 0 | 0,0 | 0 | 0,0 |
| 10 | 38569694 | 0 | 0 | 0 | 0 | 0 | 0 | 0 | 0 | 0 | 0 | 0 | 0 | 0 | 0 | 0 | 0 | 1 | 0 | 0 | 0,0 | 1 | 0,0 | 1 | 0,0 |
| 11 | 90015909 | 0 | 0 | 0 | 0 | 0 | 0 | 0 | 0 | 0 | 0 | 0 | 0 | 0 | 0 | 0 | 0 | 0 | 0 | 0 | 0,0 | 0 | 0,0 | 0 | 0,0 |
| 12 | 10253754 | 0 | 0 | 0 | 0 | 0 | 0 | 0 | 0 | 0 | 0 | 0 | 0 | 0 | 0 | 0 | 0 | 0 | 0 | 0 | 0,0 | 0 | 0,0 | 0 | 0,0 |
| 12 | 10659570 | 0 | 0 | 0 | 0 | 0 | 0 | 0 | 0 | 0 | 0 | 0 | 0 | 0 | 0 | 0 | 0 | 0 | 0 | 0 | 0,0 | 0 | 0,0 | 0 | 0,0 |
| 12 | 64115282 | 0 | 0 | 0 | 0 | 0 | 0 | 0 | 0 | 0 | 0 | 0 | 0 | 0 | 0 | 0 | 0 | 0 | 0 | 0 | 0,0 | 0 | 0,0 | 0 | 0,0 |
| 14 | 92719144 | 0 | 0 | 0 | 0 | 0 | 0 | 0 | 0 | 0 | 0 | 0 | 0 | 0 | 0 | 0 | 0 | 0 | 0 | 0 | 0,0 | 0 | 0,0 | 0 | 0,0 |
| 15 | 68449069 | 0 | 0 | 0 | 0 | 0 | 0 | 0 | nd | 0 | 0 | 0 | 0 | 0 | 0 | 0 | 0 | 0 | 0 | 0 | 0,0 | 0 | 0,0 | 0 | 0,0 |
| 17 | 25287188 | 0 | 0 | 0 | 0 | 0 | 0 | 0 | 0 | 0 | 0 | 0 | 0 | 0 | 0 | 0 | 0 | 0 | 0 | 0 | 0,0 | 0 | 0,0 | 0 | 0,0 |
| 17 | 58095893 | 0 | 0 | 0 | 0 | 0 | 0 | 0 | 0 | 0 | 0 | 0 | 0 | 0 | 0 | 0 | 0 | 0 | 0 | 0 | 0,0 | 0 | 0,0 | 0 | 0,0 |
| 18 | 26964615 | 0 | 0 | 0 | 0 | 0 | 0 | 0 | 0 | 0 | 0 | 0 | 0 | 0 | 0 | 0 | 0 | 0 | 0 | 0 | 0,0 | 0 | 0,0 | 0 | 0,0 |
| 21 | 24405628 | 0 | 0 | 0 | 0 | 0 | 0 | 0 | 0 | 0 | 0 | 0 | 0 | 0 | 0 | 0 | 0 | 0 | 0 | 0 | 0,0 | 0 | 0,0 | 0 | 0,0 |
| 22 | 19780291 | 0 | 0 | 0 | 0 | 0 | 0 | 0 | 0 | 0 | 0 | 0 | 0 | 0 | 0 | 0 | 0 | 0 | 0 | 0 | 0,0 | 0 | 0,0 | 0 | 0,0 |

**Supplementary Table S6**. Verification results of medium-confidence somatic single-nucleotide mutation calls. Yellow background colour shows the subject (column) and chromosomal position (row) at which the mutation was suspected. For each subject, verification results are shown for blood (bl) and lung brush (br) samples, the latter is further divided into one from lower lobe followed by one from upper lobe (subjects 10 to 12). The mutation was always suspected in lower lobe brushings. Numbers in black give clear indication of the absence (variant allele frequency, VAF ≈0) or presence of one (VAF ≈0.5) variant allele at the respective position. Red colour indicates less certainty and the decision about absence or presence was not only taken by the VAF, but also by read depth and cross-sample comparisons at the respective position (see Methods). Green colour suggests a high likelihood of subclonal presence of one variant allele, but may also point to positions difficult to call if present across several subjects. nd=not determinable.

| Subject | | 1 | | 2 | | 3 | | 4 | | 5 | | 6 | | 7 | | 8 | | 9 | | 10 | | 11 | | 12 | |
| --- | --- | --- | --- | --- | --- | --- | --- | --- | --- | --- | --- | --- | --- | --- | --- | --- | --- | --- | --- | --- | --- | --- | --- | --- | --- |
| Sample | | Bl | Br | Bl | Br | Bl | Br | Bl | Br | Bl | Br | Bl | Br | Bl | Br | Bl | Br | Bl | Br | Bl | Br | Bl | Br | Bl | Br |
| 1 | 146650363 | 0 | 0 | 0 | 0 | 0 | 0 | 0 | 0 | 0 | 0 | 0 | 0 | 0 | 0 | 0 | 0 | 0 | 0 | 0 | 0,0 | 0 | 0,0 | 0 | 0,0 |
| 1 | 234411511 | 0 | 0 | 1 | 0 | 1 | 1 | 1 | nd | 1 | 1 | 1 | 1 | 0 | 0 | 1 | 1 | 1 | 1 | 0 | 0,0 | 0 | 0,0 | 1 | 1,1 |
| 2 | 134650758 | 1 | 1 | 1 | 1 | 2 | 2 | 1 | 1 | 1 | 1 | 2 | 2 | 1 | 1 | 0 | 0 | 1 | 1 | 0 | 0,0 | 0 | 0,0 | 1 | 1,1 |
| 3 | 655821 | 1 | 1 | 1 | 1 | 1 | 1 | 0 | 0 | 1 | 1 | 1 | 1 | 1 | 1 | 1 | 1 | 1 | 1 | 1 | 1,1 | 1 | 1,1 | 1 | 1,1 |
| 3 | 133442608 | 0 | 0 | 1 | 1 | 0 | 0 | 1 | 1 | 1 | 1 | 0 | 0 | 0 | 0 | 0 | 0 | 1 | 1 | 1 | 1,1 | 1 | 1,1 | 1 | 1,1 |
| 4 | 9035262 | 0 | 0 | 0 | 0 | 0 | 0 | 0 | 0 | 0 | 0 | 0 | 0 | 0 | 0 | 0 | 0 | 0 | 0 | 0 | 0,0 | 0 | 0,0 | 0 | 0,0 |
| 6 | 168563807 | 2 | 2 | 2 | 2 | 1 | 1 | 1 | 1 | 0 | 0 | 1 | 1 | 1 | 1 | 0 | 0 | 1 | 1 | 2 | 2,2 | 1 | 1,1 | 1 | 1,1 |
| 7 | 151940238 | 0 | 0 | 0 | 0 | 0 | 0 | 0 | 0 | 0 | 0 | 0 | 0 | 0 | 0 | 0 | 0 | 0 | 0 | 0 | 0,0 | 0 | 0,0 | 0 | 0,0 |
| 8 | 52059335 | 0 | 0 | 0 | 0 | 0 | 0 | 0 | 0 | 1 | 1 | 0 | 0 | 0 | 0 | 1 | 1 | 0 | 0 | 0 | 0,0 | 1 | 1,1 | 1 | 1,1 |
| 8 | 54244369 | 1 | 1 | 0 | 0 | 0 | 0 | 0 | 0 | 0 | 0 | 0 | 0 | 0 | 0 | 1 | 1 | 0 | 0 | 1 | 1,1 | 2 | 2,2 | 0 | 0,0 |
| 10 | 77340410 | 1 | 1 | 2 | 1 | 2 | 2 | 2 | 2 | 2 | 2 | 0 | 0 | 0 | 0 | 2 | 2 | 1 | 1 | 2 | 2,2 | 2 | 2,2 | 2 | 2,2 |
| 11 | 129550714 | 0 | 0 | 0 | 0 | 0 | 0 | 0 | 0 | 0 | 0 | 0 | 0 | 0 | 0 | 0 | 0 | 0 | 0 | 0 | 0,0 | 0 | 0,0 | 0 | 0,0 |
| 14 | 28309043 | 1 | 1 | 1 | 1 | 1 | 1 | 1 | 1 | 1 | 1 | 2 | 2 | 1 | 1 | 1 | 1 | 1 | 1 | 1 | 1,1 | 1 | 1,1 | 1 | 1,1 |
| 14 | 37349832 | 0 | 1 | 0 | 0 | 0 | 0 | 1 | 1 | 1 | 1 | 1 | 1 | 0 | 0 | 2 | 2 | 0 | 0 | 1 | 1,1 | 1 | 1,1 | 1 | 1,1 |
| 14 | 85878628 | 1 | 1 | 1 | 1 | 1 | 1 | 0 | 0 | 1 | 1 | 2 | 2 | 1 | 1 | 0 | 0 | 0 | 0 | 1 | 1,1 | 0 | 0,0 | 1 | 1,1 |
| 15 | 24496361 | 1 | 1 | 0 | 0 | 0 | 0 | 1 | 1 | 1 | 1 | 0 | 0 | 0 | 0 | 1 | 1 | 0 | 0 | 0 | 0,0 | 2 | 2,2 | 1 | 1,1 |
| 15 | 34916901 | 1 | 1 | 0 | 0 | 1 | 1 | 0 | 0 | 1 | 1 | 1 | 1 | 0 | 0 | 1 | 1 | 1 | 1 | 1 | 1,1 | 1 | 1,1 | 0 | 0,0 |
| 16 | 32513507 | 1 | 1 | 1 | 1 | 1 | 1 | 0 | 0 | 1 | 1 | 1 | 1 | 0 | 0 | 1 | 1 | 1 | 1 | 1 | 1,1 | 1 | 1,1 | 1 | 1,1 |
| 18 | 14861823 | 0 | 0 | 0 | 0 | 0 | 0 | 0 | 0 | 0 | 0 | 0 | 0 | 0 | 0 | 0 | 0 | 0 | 0 | 0 | 0,0 | 0 | 0,0 | 0 | 0,0 |
| 18 | 63567858 | 2 | 2 | 2 | 2 | 2 | 2 | 2 | 2 | 2 | 2 | 2 | 2 | 0 | 0 | 2 | 2 | 2 | 2 | 2 | 2,2 | 2 | 2,2 | 2 | 2,2 |
| 19 | 48462795 | 0 | 0 | 0 | nd | 0 | 0 | 1 | nd | 0 | 0 | 1 | 1 | 2 | 2 | 2 | 2 | 1 | 1 | 2 | 2,2 | 0 | 0,0 | 0 | 0,0 |
| 20 | 29606022 | 0 | 0 | 0 | 0 | 0 | 0 | 0 | 0 | 0 | 0 | 0 | 0 | 0 | 0 | 0 | 0 | 0 | 0 | 0 | 0,0 | 0 | 0,0 | 0 | 0,0 |
| 21 | 17766298 | 2 | 2 | 1 | 1 | 1 | 1 | 2 | 2 | 2 | 2 | 1 | 1 | 1 | 1 | 1 | 1 | 1 | 1 | 2 | 2,2 | 1 | 1,1 | 2 | 2,2 |
| 21 | 42824661 | 0 | 0 | 0 | 0 | 0 | 0 | 0 | 0 | 1 | 1 | 0 | 0 | 0 | 0 | 0 | 0 | 0 | 0 | 0 | 0,0 | 1 | 1,1 | 1 | 1,1 |
| 22 | 17382019 | 1 | 1 | 1 | 1 | 1 | 1 | 1 | 1 | 1 | 1 | 1 | 1 | 1 | 1 | 1 | 1 | 1 | 1 | 1 | 1,1 | 1 | 1,1 | 1 | 1,1 |
| 22 | 48465870 | 1 | 1 | 2 | 2 | 2 | 2 | 0 | 0 | 0 | 0 | 1 | 1 | 1 | 1 | 2 | 2 | 0 | 0 | 1 | 1,1 | 1 | 1,1 | 0 | 0,0 |
| X | 8387066 | 0 | 0 | 1 | nd | 1 | 1 | 1 | nd | 1 | 1 | 1 | 1 | 1 | 1 | 1 | 1 | 0 | 0 | 0 | 1,0 | 0 | 1,1 | 0 | 0,0 |
| X | 111506015 | 0 | 0 | 0 | 0 | 0 | 0 | 0 | 0 | 0 | 0 | 1 | 1 | 0 | 0 | 1 | 1 | 0 | 0 | 0 | 0,0 | 0 | 0,0 | 0 | 0,0 |

**Supplementary Table S7**. Verification results of low-confidence somatic single-nucleotide mutation calls. Yellow background colour shows the subject (column) and chromosomal position (row) at which the mutation was suspected. For each subject, verification results are shown for blood (bl) and lung brush (br) samples, the latter is further divided into one from lower lobe followed by one from upper lobe (subjects 10 to 12). The mutation was always suspected in lower lobe brushings unless at the two positions concerning subject 12, where it was predicted at both airway locations. Numbers in black give clear indication of the absence (variant allele frequency, VAF ≈0) or presence of one (VAF ≈0.5) or two (VAF ≈1.0) variant alleles at the respective position. Red colour indicates less certainty and the decision about absence or presence was not only taken by the VAF, but also by read depth and cross-sample comparisons at the respective position (see Methods). Green colour suggests a high likelihood of subclonal presence of one variant allele, but may also point to positions difficult to call if present across several subjects. nd=not determinable.

Supplementary Figures


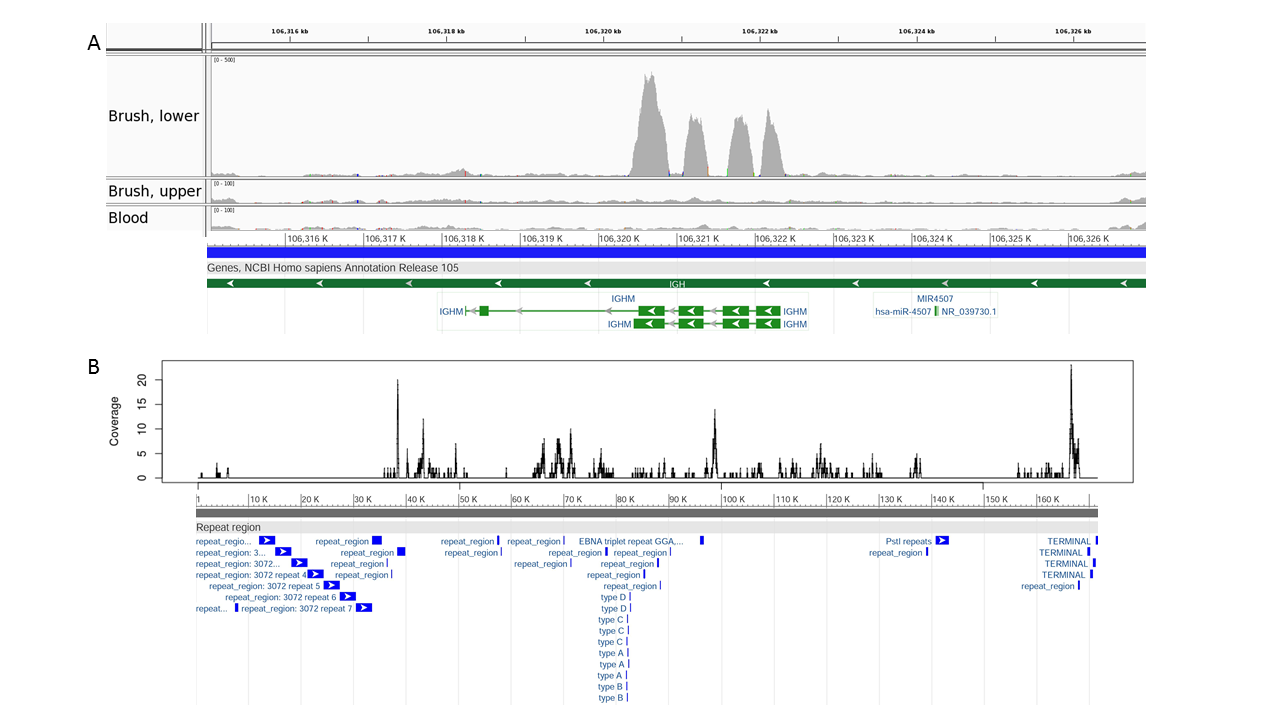
**Supplementary Figure S1**. Peculiarities in the sequencing results of the lower lobe brush sample of subject 10. (A) The *IGHM* locus showed exonic regions with strongly elevated read depth. (B) Some of the sequencing reads could be aligned to the Epstein-Barr viral genome indicating a former local infection of the airways.


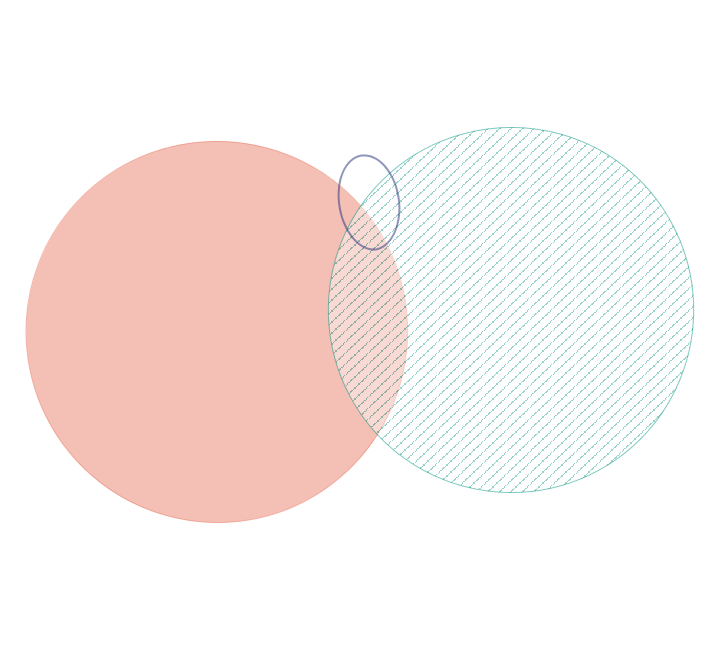

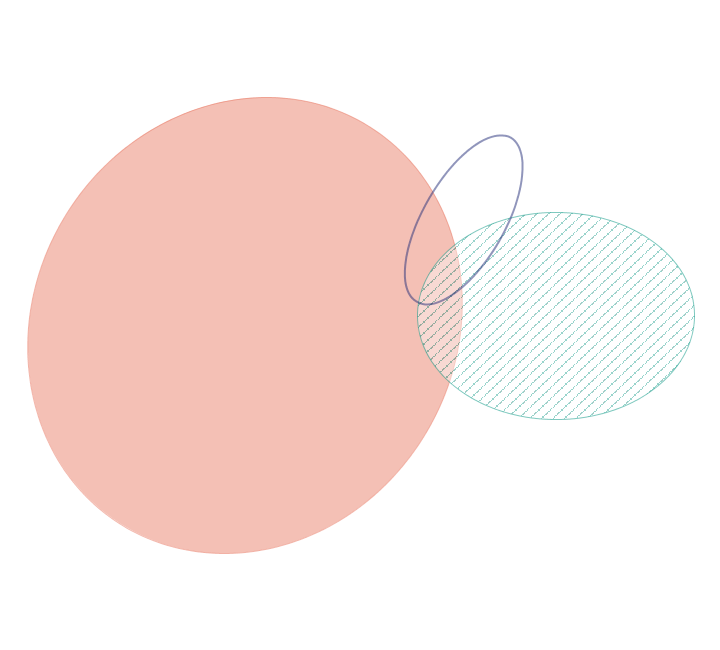

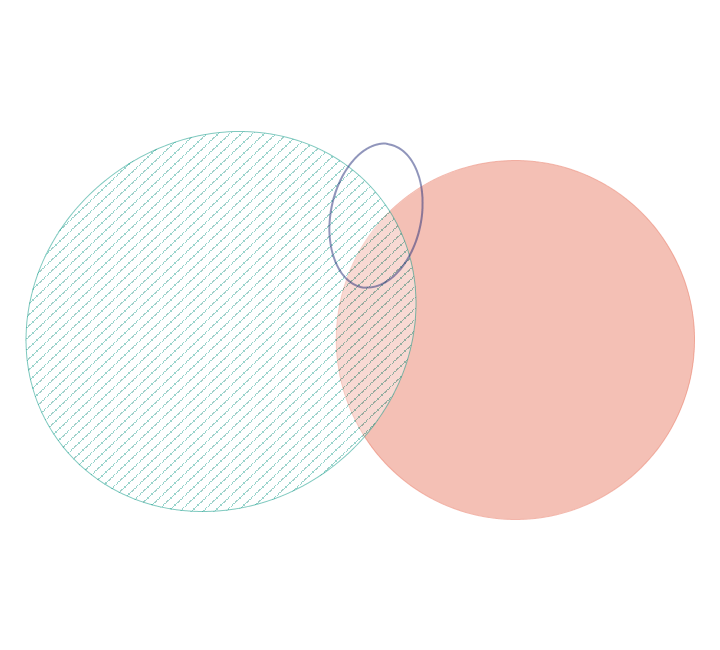

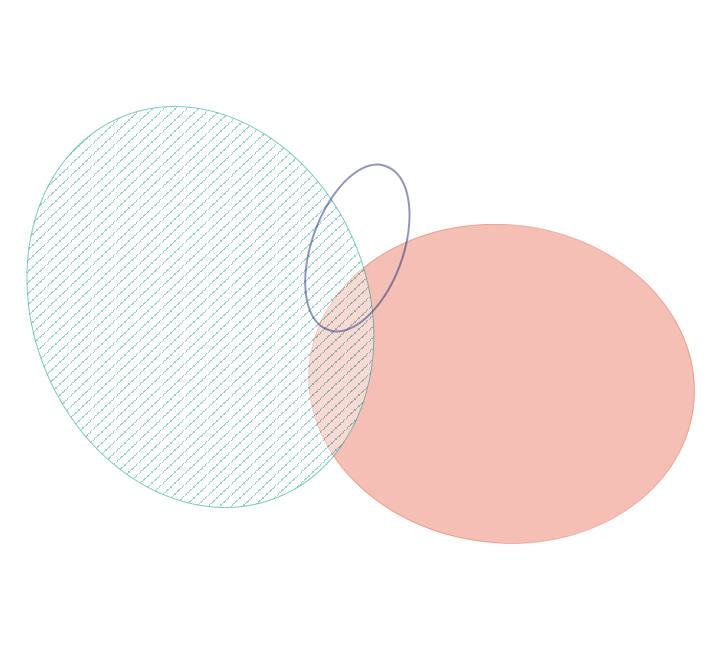

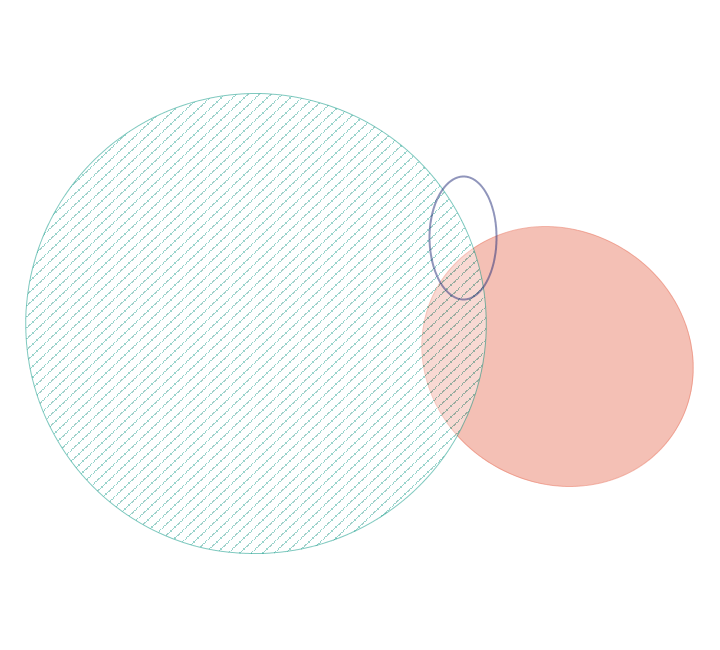

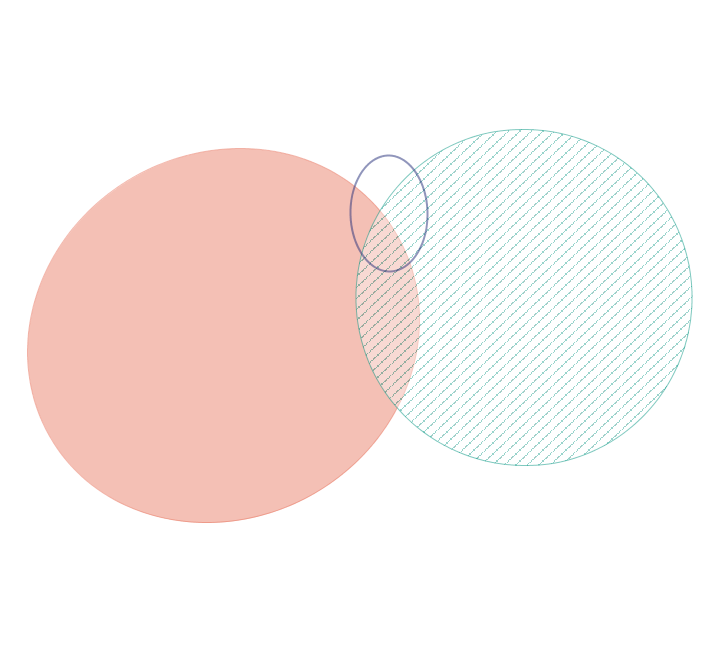

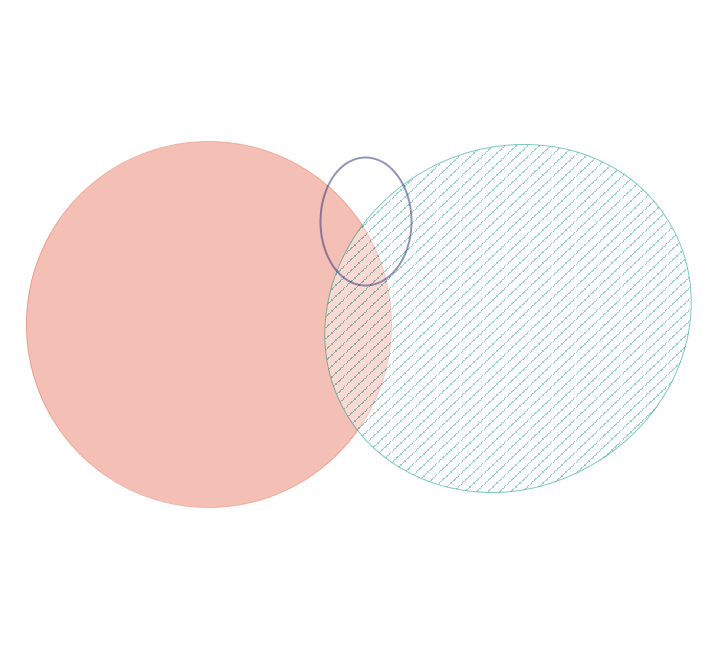

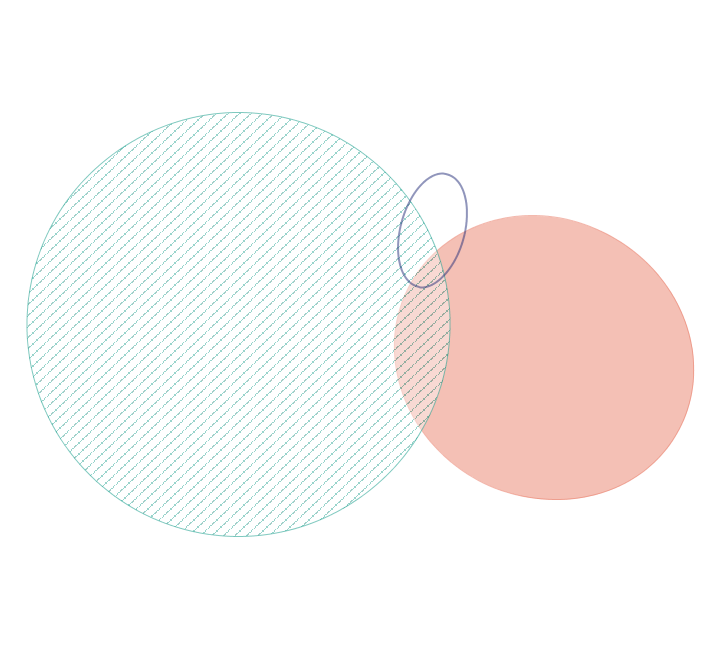

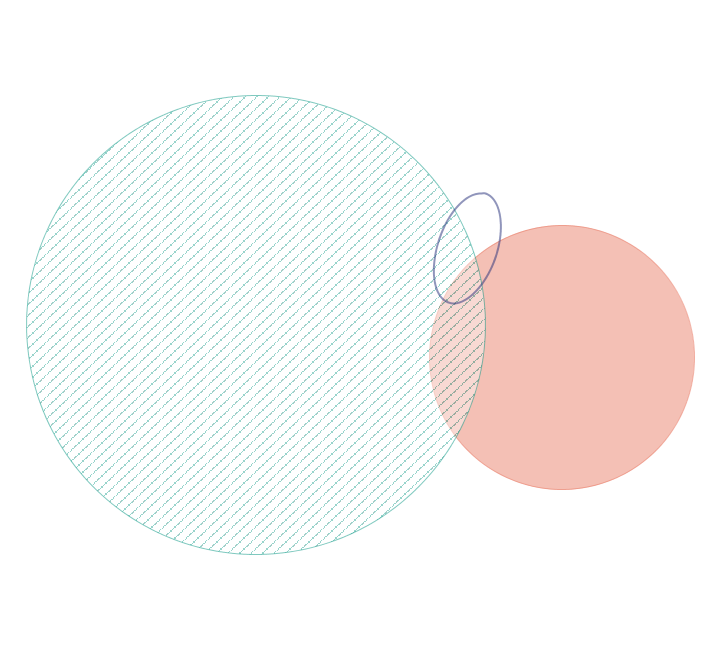

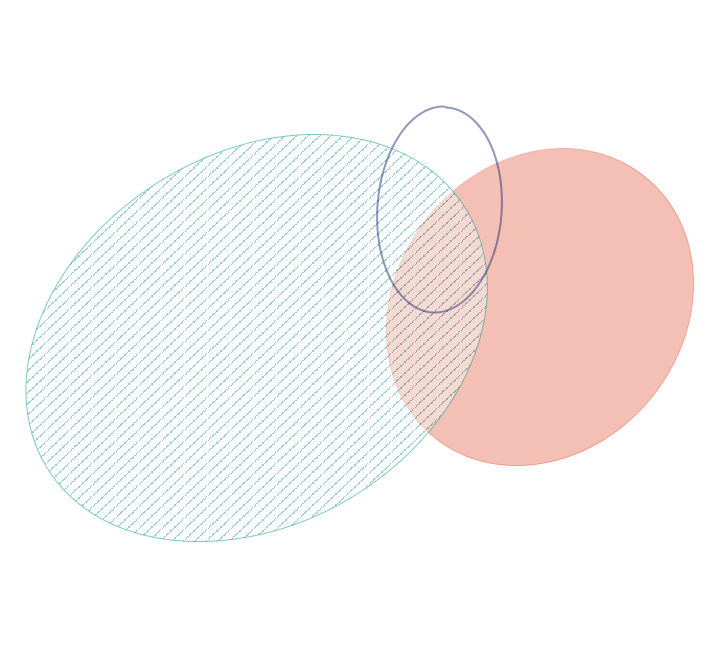

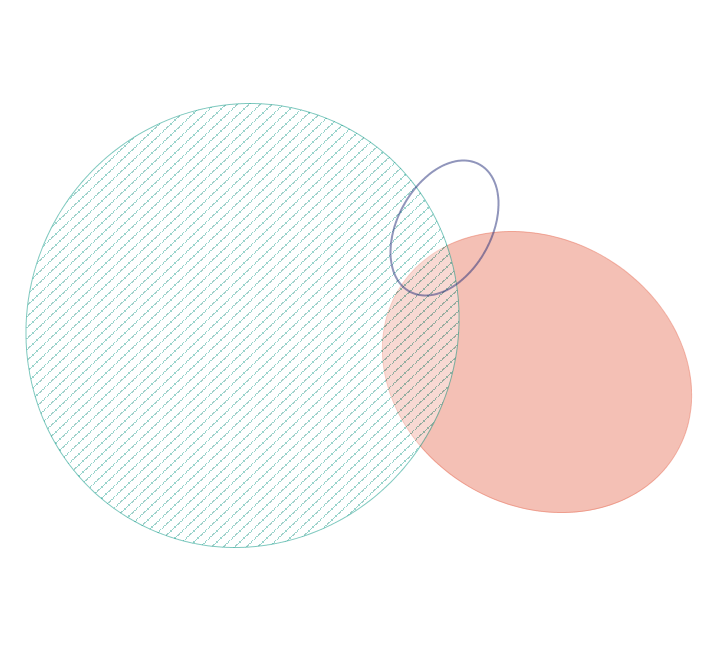

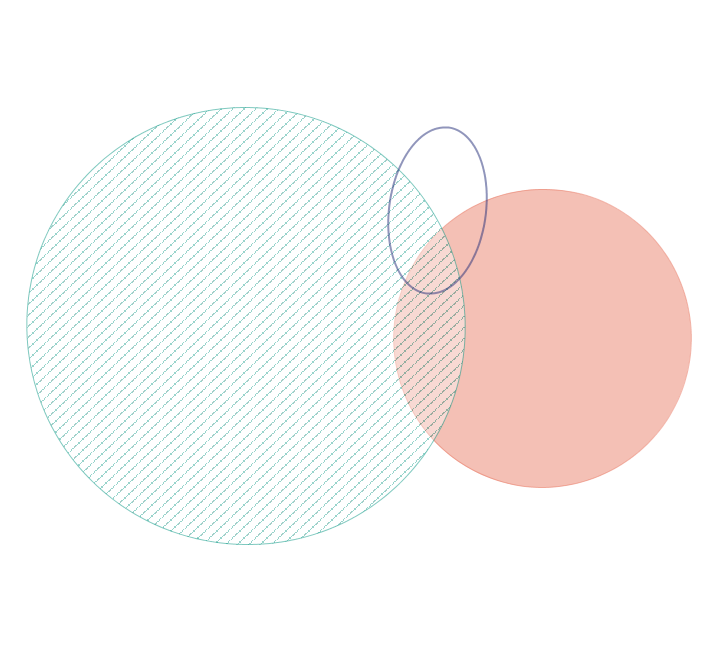

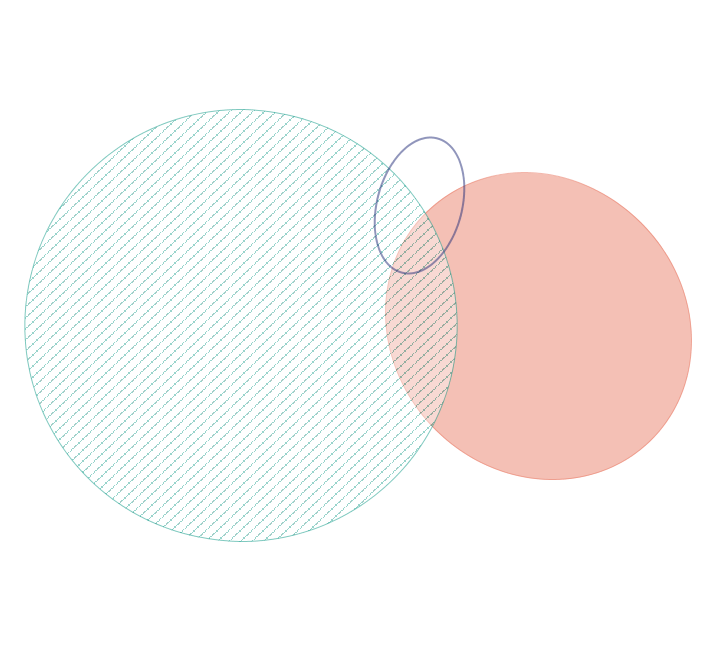

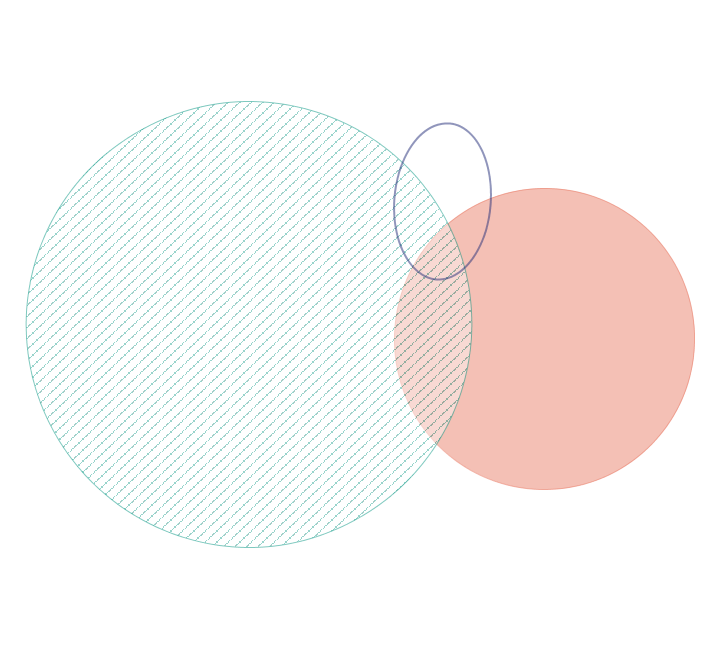

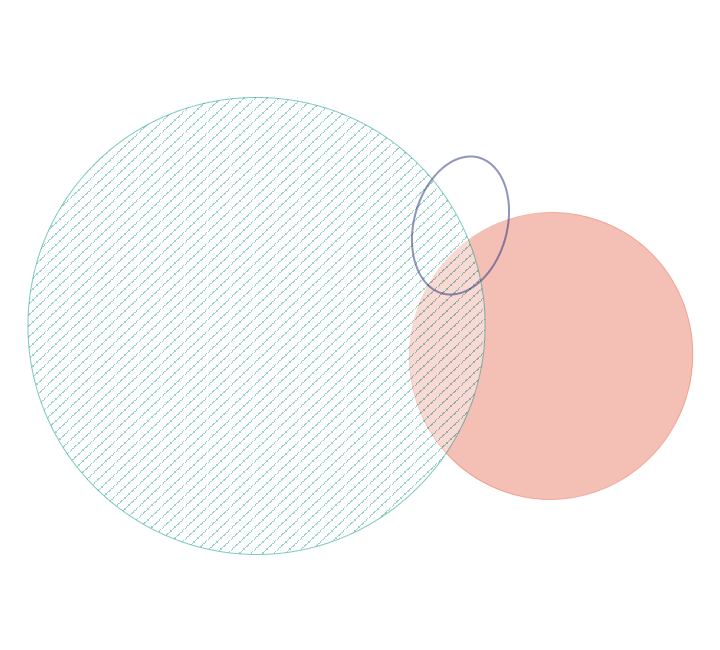


**4**

**11**

**12u**

**11u**

**10u**

**12**

**8**

**9**

**6**

**3**

**1**

**2**

**5**

**7**

**10000**

**Supplementary Figure S2**. Area-proportional Venn diagrams showing the overlap of somatic single-nucleotide mutation calls between three calling tools. MuTect (red) and VarScan 2 (green stripes) call a much larger set than Strelka (purple border). The largest set is always depicted on the left. Numbers refer to subjects and the letter “u” to results from the upper lobe brush samples.


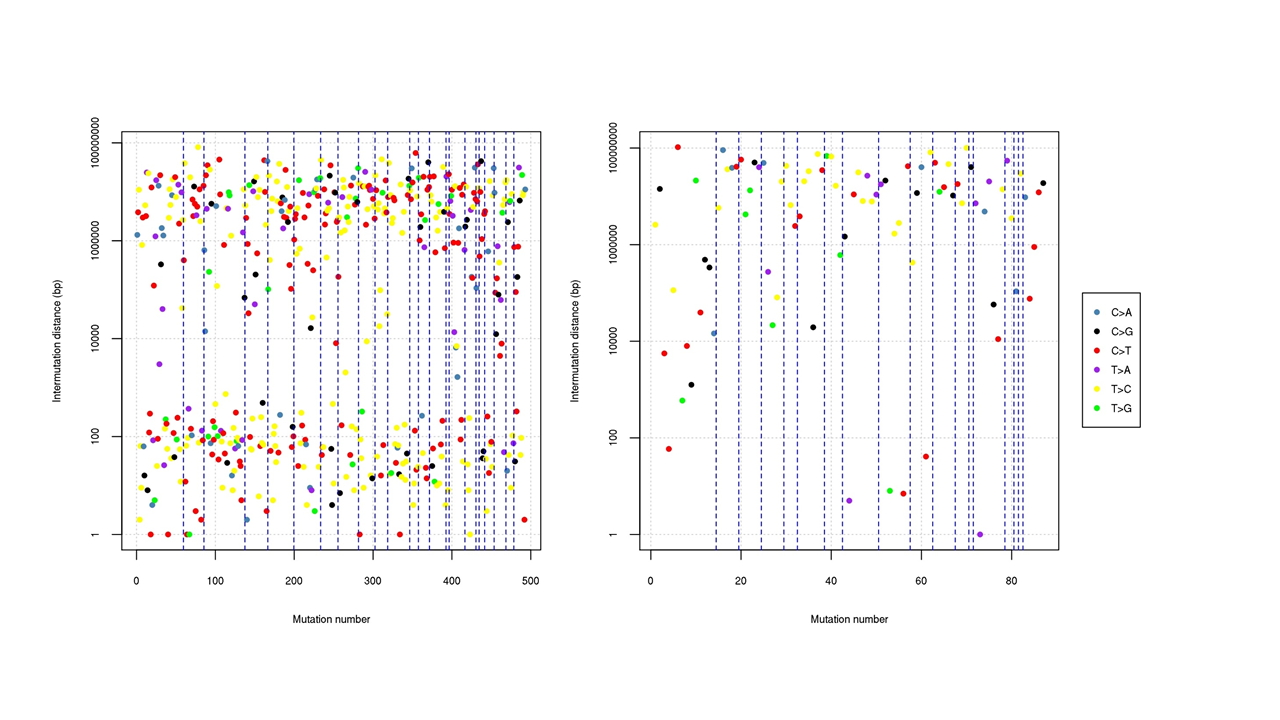


**Supplementary Figure S3**. Rainfall plots depicting intermutation distances between mutation calls in brushings of the lower lobe (left) and upper lobe (right) of subject 10. Chromosomes are shown from 1 (left) to X (right) and are separated by dashed lines. Their width is calculated according to the number of calls. No mutations were detected for chromosomes 13, 14, 19, 22 and X in the sample of the upper lobe. Colours represent the six different mutation types.


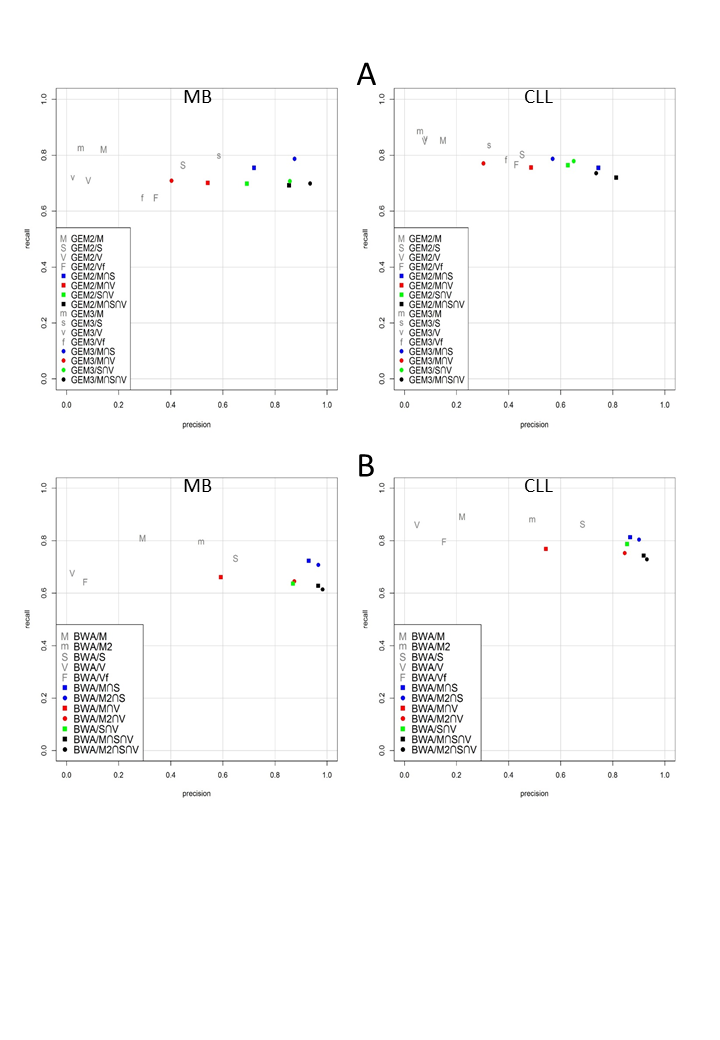


**Supplementary Figure S4**. Accuracy plots depicting different strategies to call somatic single-nucleotide mutations for MB (left) and CLL (right). (A) The updated aligning tool GEM3 was superior to GEM2 only if used in combination with Strelka and only for MB. (B) The updated mutation calling tool MuTect 2 showed higher accuracy than MuTect (in combination with BWA-mem), but improvement was marginal in the overlap with other mutation calling tools. MB=medulloblastoma, CLL=chronic lymphocytic leukemia, M=MuTect, S=Strelka, V=VarScan 2, Vf=VarScan 2 plus filter, M2=MuTect 2.
